# Graphene Nano‐Blister in Graphite for Future Cathode in Dual‐Ion Batteries: Fundamentals, Advances, and Prospects

**DOI:** 10.1002/advs.202207426

**Published:** 2023-03-22

**Authors:** Yitao He, Yujie Dong, Yaohui Zhang, Yongtao Li, Haijin Li

**Affiliations:** ^1^ Department of Energy and Power Engineering School of Energy and Environment Anhui University of Technology Ma'anshan Anhui 243002 China; ^2^ School of Physics Harbin Institute of Technology No. 92 Xidazhi Street Harbin Heilongjiang 150001 China; ^3^ Key Laboratory of Green Fabrication and Surface Technology of Advanced Metal Materials Ministry of Education Anhui University of Technology Ma'anshan Anhui 243002 China

**Keywords:** dual‐ion batteries, graphene blisters, graphene in graphite, graphite cathodes

## Abstract

The intercalating of anions into cost‐effective graphite electrode provides a high operating voltage, therefore, the dual‐ion batteries (DIBs) as novel energy storage device has attracted much attention recently. The “graphene in graphite” has always existed in the graphite cathode of DIBs, but has rarely been researched. It is foreseeable that the graphene blisters with the intact lattice structure in the shell can utilize its ultra‐high elastic stiffness and reversible lattice expansion for increasing the storage capacity of anions in the batteries. This review proposes an expected “blister model” by introducing the high elasticity of graphene blisters and its possible formation mechanism. The unique blisters composed of multilayer graphene that do not fall off on the graphite surface may become indispensable in nanotechnology in the future development of cathode materials for DIBs.

## Graphical abstract

The intercalation of solvated anions enables a few graphitic layers to separate from the bulk graphite and form a nano‐blister. The number of graphite layers in the blister shell is often less than 10, and the physical properties of blister shell similar to those of graphene make it different from bulk graphite, so it can be referred as “graphene blister”.

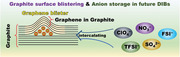

## 1 Introduction

**Figure 1:**
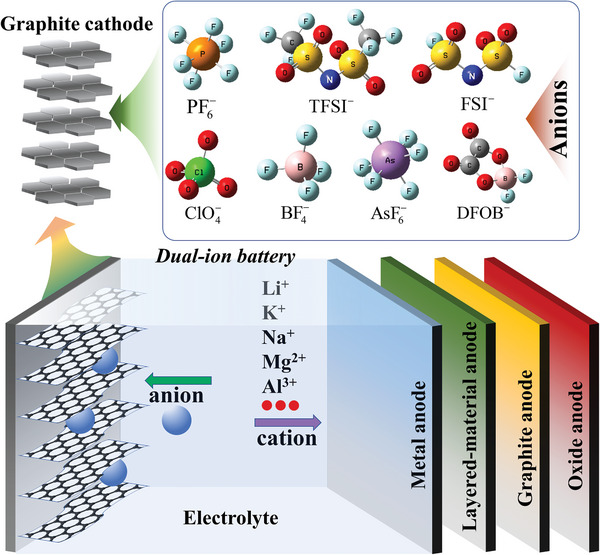
Schematic illustration of a dual‐ion battery.

DIBs are considered as a promising energy storage device wherein anions and cations are intercalated/de‐intercalated into the cathodes and anodes, respectively.^[^ ^1^ ^]^ Compared with widely used lithium‐ion batteries (LIBs), DIBs have greater potential for large‐scale energy storage applications owing to the higher operation voltage caused by the intercalation/de‐intercalation of anions into/out of the cathode.^[^ ^2^ ^]^ Additionally, the electrode materials in DIBs are predominantly carbon materials (such as graphite); when graphite is used for the anode and cathode, the DIBs can also be referred to as “dual‐graphite batteries” rather than transition metal‐based materials, presenting great advantages of DIBs such as long cycling‐life, environmental protection, inexpensiveness, and high energy density.^[^ ^3^ ^]^ A schematic illustration of a DIB is shown in **Figure** **1**.

**Figure 2:**
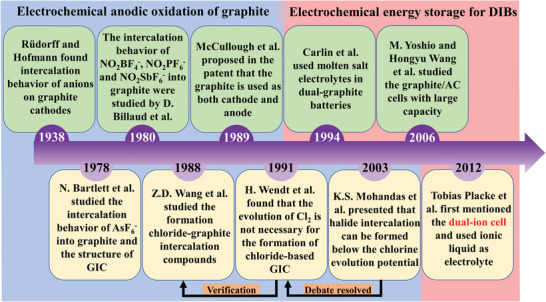
A brief development history of DIBs until the device has a unified name.

Although DIBs have been studied since 1938,^[^ ^4^ ^]^ researchers explored the structure and composition of various graphite anion intercalation compounds (GICs) primarily through graphite oxidation in the early stage. A brief development history of DIBs until the device has a unified name have been provided in an image (**Figure** **2**). In the oxidation experiments, the highly oriented pyrolytic graphite (HOPG) was often used as an intercalation host for anions because of its large size and high graphitization degree. Such intercalated anions include AsF_6_^−^,^[^ ^5^ ^]^ BF_4_^−^, PF_6_^−^, and SbF_6_^−^.^[^ ^6^ ^]^ Meanwhile, there was an academic debate over the existence of stable chloride GICs in the graphite lattice.^[^ ^7^ ^]^ This debate was not resolved until 2003, when the specific characteristics of electrochemical Cl^−^ anion intercalation into graphite were studied by Mohandas et al. They concluded that the potential at which chloride‐GICs could be formed was below that required to produce Cl_2_ gas.^[^ ^8^ ^]^ These results provide a theoretical basis for DIBs utilization of halide anions, such as halogen conversion intercalation aqueous battery.^[^ ^9^ ^]^ Moreover, another DIB research focused on the influences of various electrolytes, such as molten salt electrolytes or organic electrolytes, on the intercalation/de‐intercalation process and the electrochemical DIB performance.^[^ ^10^ ^]^ In the study on organic electrolytes, Yoshio and Wang conducted numerous studies on the influences of solvents and anions on the intercalation behavior. In 2006, they used artificial KS6 graphite and active carbon (AC) as the cathode and anode, respectively, for assembling the DIBs, and a solution of 1 m BF_4_^−^ in PC was used as the organic electrolyte. Although traditional carbon‐based supercapacitors possess high power density, they often possess low energy density. However, the combination of graphite and AC electrodes could significantly improve the energy density of the devices and was known as “megalo‐capacitance” capacitor.^[^ ^11^ ^]^ Although the name of this energy storage device varied, the most significant feature of this battery type is the reversible intercalation/de‐intercalation reaction of anions into/out of the graphite cathode. The device did not have a unified name until 2012, when the name of “dual‐ion cell” was first mentioned by Tobias et al.^[^ ^12^ ^]^ Furthermore, Wang et al. attached significant importance to the research on the solvation effect, and proposed that the electrolyte significantly influences the performance of this type of capacitor. The charge‐storage behavior can be regarded as the competition between the electrode and electrolyte for acquiring anions; thus, anion solvation could play a key role in the anion intercalation into carbonaceous materials. For example, the interactions between the PF_6_^−^ anion and EC or PC solvent were explored earlier, and the results demonstrated that the solvation effect grew stronger with decreasing anion size. Therefore, the strong interaction between PF_6_^−^ and EC hinders the PF_6_^−^ intercalation into graphite.^[^ ^13^ ^]^ In addition, the influences of EC, PC, and GBL solvents on the intercalation behavior of BF_4_^−^ anion with a small size were also well summarized.^[^ ^14^ ^]^ Owing to the strong bonding between EC and BF_4_^−^, the EC solvent significantly shortened the discharge plateau and specific capacity.

Previous studies have focused on i) the GIC structure in graphite electrodes formed by electrochemical anion intercalation, and ii) the influence of the solvated anion composition on battery capacity and cycle life. However, these two research directions were relatively separated, and the electrolyte as the only source of anions and the graphite as the anion host should be simultaneously focused on. From this point of view, methods to identify hidden key research priorities must be explored to guide and promote the future development of DIBs.

As an important electrochemistry research topic, the electrode/electrolyte interface affects the properties of ion adsorption, redox reaction, and desorption.^[^ ^15^ ^]^ Therefore, the interface properties optimization is required to improve the electrochemical performances of DIBs.^[^ ^16^ ^]^ In recent years, the regulation of electrolyte components on the graphite cathode interface in DIBs has gradually attracted attention.^[^ ^17^ ^]^ This method, via the regulation of CEI or SEI composition in Li‐ion or Li‐metal batteries, has been demonstrated to be effective to an extent. For example, the oxidative decomposition of the electrolyte was suppressed, and the structural integrity of graphite was maintained by regulating the CEI composition at the graphite interface using fluorinated solvent.^[^ ^18^ ^]^ However, the special 2D structure of graphite differ significantly from those of other cathode materials.^[^ ^19^ ^]^ In‐depth research on the interface properties of graphite cathodes remains lacking, and the influence of anion intercalation on the graphite surface properties in DIBs also required to be further studied.

Blister formation on graphite surfaces was a relatively common phenomenon observed in the field of graphite electrochemical oxidation.^[^ ^20^ ^]^ Graphite surface blistering was considered to be the initial stage of graphite exfoliation and has attracted the attention of several researchers.^[^ ^21^ ^]^ Similarly, the graphite cathode of the DIBs underwent the same electrochemical oxidation process.^[^ ^22^ ^]^ However, significant information on graphite surface blistering remain uncovered. Once the blisters are formed, multi‐layer graphite that was originally closely connected to the bulk graphite, turns into bended graphene. The number of graphite layers in the blister shell is often less than 10 layers, and the physical properties of blister shell similar to those of multilayer graphene make it different from bulk graphite, so we can refer to it as “graphene blister.” It has been reported that this type of blister generated during the charge/discharge process of a graphite cathode is beneficial for improving the specific capacity of DIBs.^[^ ^23^ ^]^ However, research specifically focusing on the contribution of graphene blisters to the DIB performance remains lacking. It is foreseeable that the graphene blisters intact lattice structures in the shell can utilize ultra‐high elastic stiffness and reversible lattice expansion for increasing the anion storage capacity in the batteries.

In this review, therefore, the combinations of reported results from many different research fields, including graphite oxidation, nano‐mechanics of graphene, condensed‐matter physics, electrowetting, and DIBs, are presented for sorting out which supports intercalation of anions and formation of graphene blister in the bulk graphite. Besides, we discussed the reversible deformation and anion storage capacity of graphene blisters from the elasticity of graphene blisters and their relationship with graphite grain boundaries. A “blister model” was proposed by linking the many characterizations and analyses in the literatures. The conclusions of the model were applied to explain many anomalous behaviors in DIBs. The revelation of the importance of “graphene in graphite” for anion intercalation can develop future research propositions that can extend the field of DIBs.

## 2 Overview of Graphene Blister

### 2.1 Research on Graphene Blister

**Figure 3:**
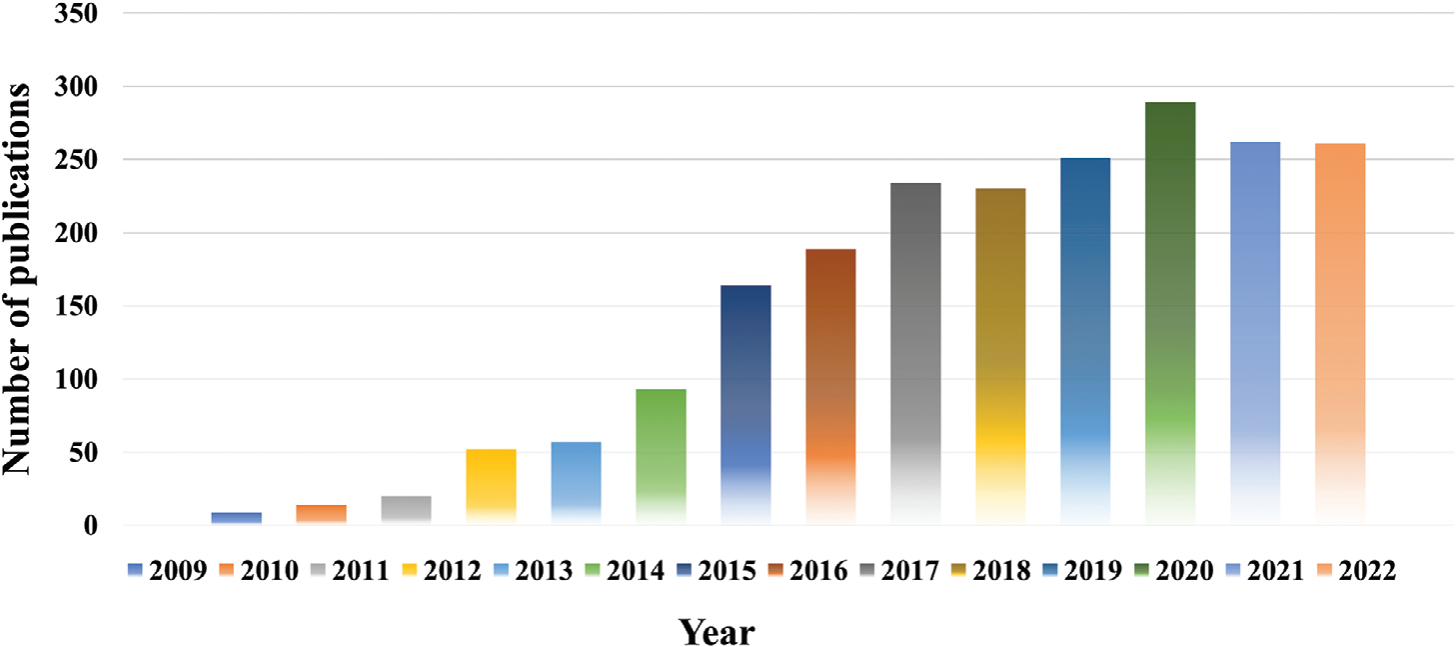
Number of publications related to graphene blister from 2009 to 2022 (data extracted from Web of Science).

Although graphene was discovered in 2004, graphene blisters with multilayer formed on the graphite surface were researched much earlier. The graphene blisters are a kind of microscopic 3D network nanostructures with a series of special properties. Benefiting from their ultrahigh adhesion, ultrahigh elasticity and excellent electrical conductivity, graphene blisters gather plenty of attractive physical properties. Dresselhaus and Kalish prepared carbon materials with specific properties through ion implantation, which induced modifications to graphite like blistering.^[^ ^24^ ^]^ Thought provokingly, Tatsumi et al. found that introduction of nano‐sized pore into graphene sheet could provide a large capacity of 147 mAh g^−1^ through intercalation of PF_6_^−^.^[^ ^25^ ^]^ Moreover, the blistering on the graphite surface was also regarded as a necessary pre‐step in electrochemical methods to prepare graphene. Therefore, many research studies on graphene blisters or bubbles have been conducted by scientists in a variety of fields, and the researches related to graphene blisters were growing in decades as shown in **Figure** **3**. Evidence is mounting that graphene blisters in graphite electrodes play an important role in improving ion storage capacity. This review may arouse the emotions of researching graphene blisters in the field of energy storage nano‐sized materials, especially for the electrode materials in DIBs.

### 2.2 Structure and Morphology of Graphene Blister

**Figure 4:**
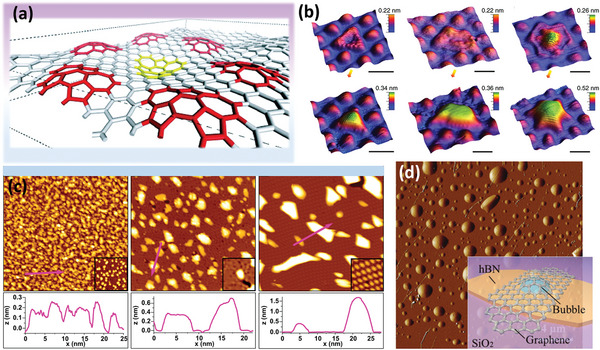
The structural representations of graphene blisters: a) A blister pushed down rather than up with packed metacrystal. Reproduced with permission.^[^ ^26^ ^]^ Copyright 2008, American Physical Society. b) 3D scanning tunneling microscopy (STM) images of triangular, trapezoidal, and hexagonal graphene blisters (the scale bars are 3 nm). Reproduced with permission.^[^ ^27^ ^]^ Copyright 2012, Macmillan Publishers Limited. c) STM topographs of graphene/Ir(111) and associated height profiles of blisters after exposure to Xe^+^ ion flow at different temperatures. Reproduced with permission.^[^ ^28^ ^]^ Copyright 2015, American Physical Society. d) AFM image of spontaneous blisters formed by graphene sheets (Inset: Schematic diagram of multilayer graphene on hBN/SiO_2_/Si substrate). Reproduced with permission.^[^ ^29^ ^]^ Copyright 2022, John Wiley & Sons.

There are several physical and chemical environments among various forms in which the graphene blister might exist. A monolayer or multilayer graphene blister forms on graphite surface or other substrates, such as SiO_2_, while the blister is not completely detached and keeps intact lattice. The structural representations of graphene blisters in many forms are shown in **Figure** **4**.

### 2.3 Properties of Graphene Blister

Elasticity, adhesion, flexility are particularly important physical parameters in graphene blisters. The excellent elasticity can facilitate the repeated or even reversible changes of the blister shell. The ultrahigh adhesion between graphene blister and substrate enable repairability or even recyclability after blister generation, and the blister is restricted to the bulk materials that adhere to the substrate surface. Graphene blisters with hollow space are more appropriate for storage of ions in electrolyte due to reversible changes of the blister shell with intact lattice. More importantly, the elastic structure of blisters can be tailored by electrochemically permeating the ion species or regulating the voltage between the graphite electrode and electrolyte to better match DIBs in terms of surface chemistry.

## 3 Formation of Graphene Blister through Electrochemical Intercalation of Anions and Their Special Properties

**Figure 5:**
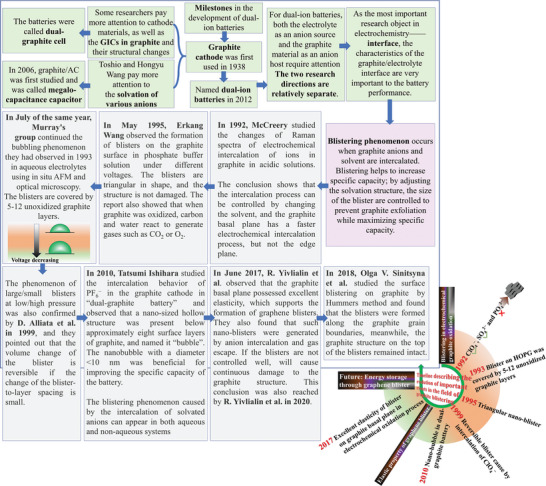
An image describing the evolution of important events in the field of graphite blistering since its discovery.

In the field of oxidation or electrochemical oxidation of graphite, anion/solvent intercalation was a crucial step in graphite exfoliation, during which nano/micro‐sized blisters were observed on the graphite basal plane.^[^ ^30^ ^]^ Once the blisters are formed, the multi‐layer graphite that was originally closely connected to the bulk graphite transforms into bended graphene.^[^ ^31^ ^]^ Similar blisters that are beneficial for improving the capacity have also been observed on the graphite cathode surface. Therefore, this section aims to analyze the graphene blister as a special phenomenon that has been ignored in DIBs for a long time. Herein, we describe this graphene blister in detail and predict its role in the future development of DIBs. The evolution of important events in the field of graphite blistering since its discovery was organized into an image as shown in **Figure** **5**. First, because DIBs utilize the electrochemical oxidation of the graphite cathode to achieve energy storage, we can elucidate the nature of the graphene blister that forms in DIBs by combining the results in the field of electrochemical graphite oxidation.

### 3.1 Blisters Formed on Graphite via Anion Intercalation

**Figure 6:**
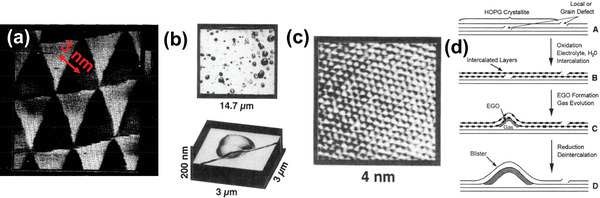
a) A STM image of superperiodic features within the anodized domain on HOPG surface. Reproduced with permission.^[^ ^34^ ^]^ Copyright 1995, Elsevier B.V. b) Ex situ AFM images of an oxidized HOPG electrode obtained in air. Upper figure: 14.7 × 14.7 µm region with many small blisters; bottom figure: 3 × 3 µm region illustrating blister with defect running continuously up and over it. Electrode oxidized by four potential cycles between 0.1 and 1.62 V; total anodic charge density/monolayer equivalent charge = 3.70. c) 4 × 4 nm atom‐resolved image obtained on top of a blister. Reproduced with permission.^[^ ^35^ ^]^ Copyright 1993, American Chemical Society. d) Schematic diagram of blister formation model proposed by Murray's group. Reproduced with permission.^[^ ^36^ ^]^ Copyright 1995, American Chemical Society.

The blistering phenomenon on the graphite surface was discovered in the 1990s, when researchers studied the electrochemical graphite oxidation in electrolytes.^[^ ^32^ ^]^ In 1992, in a study on the electrochemical intercalation process of ClO_4_^−^, SO_4_^2−^, and PO_4_^3−^ anions into graphite in an acidic aqueous solution and the corresponding Raman spectral changes during the process, McCreery et al. observed whether the graphite lattice was destroyed was correlated with the different types of anions and solvents.^[^ ^33^ ^]^ The results demonstrated that PO_4_^3−^ could not intercalate, and no lattice damage in graphite was observed. Anion intercalation was accompanied by lattice damage in both 1 m HClO_4_ and 1 m H_2_SO_4_ aqueous solutions, whereas in NaClO_4_/CH_3_CN electrolyte, ClO_4_^−^ could intercalate into the graphite lattice without lattice damage. Particularly, the rapid electrochemical intercalation process occurred on the graphite basal plane rather than the edge plane. These results indicate that the intercalation process can be controlled by regulating the anion and solvent compositions, and the anion intercalation sites cannot increase merely by increasing the fracture exposure. In 1995, Erkang Wang et al. used STM to study the electrochemical oxidation of HOPG. They observed the blister formation on the graphite surface in the phosphate buffered solution under different voltages. The blisters were triangular in shape, and the graphitic structure remained intact, indicating that intercalated ions or solvents were present under the subsurface, as shown in the bright triangle in **Figure** **6**a. However, according to previous studies, phosphate anion cannot intercalate into graphite. Therefore, the blisters observed by Wang et al. on the graphite surface were likely caused by the intercalation of water molecules, which also indicated that solvent molecules can intercalate and induce blistering phenomenon.^[^ ^34^ ^]^ This type of blister was characterized by its small size, and the STM image showed that the distance between the centers of the dark and light triangles was merely 3 nm. The size and shape of the blisters were uniformly distributed periodically. In addition, the authors believed that the intercalation sites were located one or two graphite layers below the surface layer, and the graphite defects were beneficial to the blister formation.

In the same year, Murray et al. continued studying the blistering phenomenon on the HOPG surface that they discovered in 1993 using an in situ atomic force microscopy‐electrochemistry (AFM‐EC) system and optical microscopy in aqueous electrolytes.^[^ ^35^, ^36^ ^]^ They performed electrochemical oxidation of graphite cathode in the 1 m LiClO_4_, 1 m (NH_4_)_2_SO_4_, 1 m HNO_3_, and 1 m H_2_SO_4_ aqueous solutions, respectively, and observed that the anion intercalation into graphite could lead to blistering, whereas no blistering was observed in the 1 m K_2_HPO_4_ and 1 m H_3_PO_4_ electrolytes owing to the lack of intercalation of phosphate ions. The energy‐dispersive X‐ray elemental microanalysis (EDX) results demonstrated that graphite oxide was present on the top surface of the inner blister, which was covered by 5–12 unoxidized graphite layers. The average size of semicircular blisters observed was larger than that of triangular blisters, as shown in Figure 6b. When the top surface of the outer blister was observed under high‐resolution STM, the image showed a normal hexagonal pattern with a periodic side length of 2.5 (±0.01) nm, which was similar to that of basal plane on pristine graphite (2.46 Å), indicating that the graphitic structure on the topmost surface was not damaged (Figure 6c). These conclusions were consistent with the finding of Wang. The authors further proposed that the blister formation and graphite structure destruction were two different reaction mechanisms. Moreover, according to the experimental results, the authors also suspected that the blistering phenomenon was related to the water solvent and gas evolved at the graphite electrode. Combined with the observations, the detailed process of blister formation was explained as follows: when intercalation occurs at a high voltage, the blister is small, but gas is generated, whereas during de‐intercalation, gas overflow increases the blister size. In this model, the blistering is a delamination process driven by the mechanical stress of subsurface oxidation with gas formation,^[^ ^36^ ^]^ as shown in Figure 6d. Additionally, we can propose that the triangular blisters can only be observed under high‐resolution microscopy in the nano‐size scale, and the larger blisters become hemispherical bulges.

**Figure 7:**
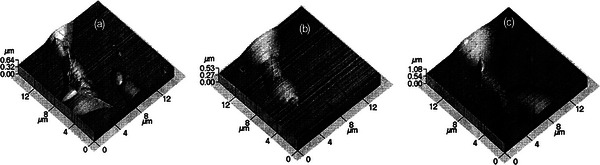
Blister formation on a HOPG surface in 2 m HClO_4_ under different potentials: a) 0 V; b) 0.95 V; c) 0 V. Reproduced with permission.^[^ ^37^ ^]^ Copyright 1999, American Chemical Society.

This same phenomenon was also observed by Alliata et al. in 1999 when they studied the intercalation behavior of the ClO_4_^−^ anion in HOPG.^[^ ^37^ ^]^ The results demonstrated that during the charge/discharge cycle (non‐first), two groups of blisters were distinctly observed at 0 V (**Figure** **7**a). When the potential was shifted to +0.95 V (Figure 7b), the smaller group of blisters and a part of the larger blisters disappeared, but when the potential was shifted back to 0 V (Figure 7c), the disappeared blister group reappeared, while few blisters merged. This phenomenon appears to be inconsistent with the conventional electrochemical processes, wherein the high potential increases blister sizes owing to an increased amount of intercalated anion, but the experimental observations are quite the opposite. Moreover, the main conclusions drawn from the research are that: i) The graphitic expansion in the form of blister formation is reversible if the intercalation leads to a small interlayer expansion (<18.7%). Results from another experiment by the authors showed that the blisters caused by intercalation of ClO_4_^−^ into graphite were reversible at low voltages;^[^ ^38^ ^]^ ii) The interlayer expansion caused by anion intercalation caused mechanical deformation of the graphite layer, and the energy required to overcome the deformation increased with the increasing number of graphite layers on both sides of the intercalation point. Therefore, the intercalation generally initiates near the surface and then proceeds into a deeper interlayer. The intercalation reaction for the solvated anion could be described by:

(1) $$\left[c_{x}\right]+\left[A^{-}\right]+y\left(\mathrm{solv}\right)=\left[c_{x}^{+}A^{-}{\left(\mathrm{solv}\right)}_{y}\right]+e^{-}$$

where c represents the carbon in graphite; *x* is the number of carbon atoms involved in the reaction in graphite; A^−^ represents the anion; solv represents the solvent molecules in the solvation shell; *y* is the number of solvent molecules involved in the intercalation reaction.

**Figure 8:**
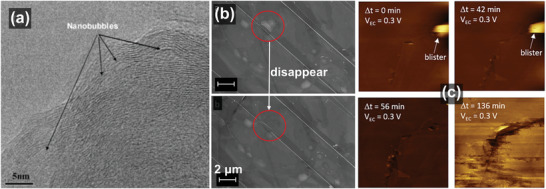
a) TEM image of (002) plane of the graphitic carbon after application of 3.1 V. Reproduced with permission.^[^ ^23^ ^]^ Copyright 2010, Elsevier B.V. b) SEM images of the graphite surface after the intercalation stage‐III: The same surface region (acquired about 1 min later with respect to the previous panel) before and after the “disappearance” of a blister. c) In situ EC‐AFM images (4 × 4 µm^2^) acquired at 0.3 V after the voltage extended up to the intercalation stage‐III (1.0 V). Reproduced with permission.^[^ ^39^ ^]^ Copyright 2017, American Chemical Society.

Because the electrochemical graphite oxidation occurs at the cathode of the DIB during charging, the blisters have been also observed in the study of DIBs. In 2010, Ishihara et al. studied the intercalation behavior of PF_6_^−^ in the graphite cathode in “dual‐graphite battery” and observed that a nano‐sized hollow structure was present below ≈8 surface layers of graphite, and named it “bubble” (**Figure** **8**a).^[^ ^23^ ^]^ The number of graphite layers constituting the bubble shell is consistent with the research results of Murray et al. (5–12 layers), and this bubble is the same as the aforementioned blister. Furthermore, the authors also observed that this nanobubble with a diameter <10 nm was beneficial for improving the specific capacity of the battery. A non‐aqueous solvent (electrolyte: 1 m *n*‐BPPF_6_ in PC/MEC) was used in this battery, which complements the speculation of the previous researchers that blistering is related to water molecules in an aqueous solution, indicating that the blistering phenomenon caused by the intercalation of solvated anions can appear in both aqueous and non‐aqueous systems. However, such bubbles (or blisters) in DIBs have not received significant attention and were only considered as lattice expansions due to anion intercalation.

There appears to be little disagreement regarding whether blisters are sufficiently stable and whether blister formation is reversible. In 2017, Yivlialin et al. used advanced microscopy techniques to analyze the structural decomposition and exfoliation of graphite during the electrochemical oxidation process.^[^ ^39^ ^]^ They observed that the graphite basal plane possessed excellent elasticity, and was morphologically stable in the electrolyte under low voltages. The anions cannot form the stage‐III structure in the graphite, while the graphite surface was not damaged. After further increasing the voltage, although the larger potential caused cracks on the graphite surface, the blisters predominantly remained stable. Furthermore, when the blisters were exposed to the high accelerating voltage probe of the scanning electron microscope (SEM), the blisters tended to disappear or “deflate” (Figure 8b, 1 minute test interval between the 2 images). The authors provided a possible explanation that the gases generated during anion intercalation were trapped inside the graphitic layered structure could escape through the defects on the basal plane. Moreover, the D peak, indicative of significant structural disorder, measured on the blister surface was absent in the experimental Raman spectrum, indicating that the blister surface produced by electrochemical oxidation exhibits no lattice damage at the nanoscale. However, on the continuous application of high voltage to the graphite electrode, the authors observed the same phenomenon of the sudden disappearance of the blister as shown in the SEM (Figure 8c). The authors explained that although the blister is stable under some conditions, a critical point exists. Once the elasticity of the blister is in sufficient to accommodate the excess materials brought by the high voltage, and a few difficult‐to‐observe channels appear, thereby releasing the internal gas, and subsequently causing the sudden blister disappearance. In this study, the authors mentioned the keyword “elastic deformation” for the first time, and emphasized that the graphite surface appears to possess some elasticity. The elasticity is an important physical basis for blister formation. If the elastic deformation is not terminated in time after reaching the limit, the graphite structure of the blister shell is subject to continuous damage.

According to a structured literature review in this section, a class of univalent ions, such as ClO_4_^−^ and PF_6_^−^, allows for easier intercalation, while it is challenging for multivalent ions, such as PO_4_^3−^, to be intercalated. The blister formation in the graphite subsurface is also predominantly related to the intercalate‐able ion ClO_4_^−^ or PF_6_^−^, because the solvent molecules can co‐intercalate with this type of anion into the graphite interlayer. The solvent molecules undergo oxidation reactions at the graphite electrode to generate gas, which subsequently generates blisters when the gas escapes during discharge. There are two possible outcomes for the formation of these blisters: The exfoliation of the graphitic structure or accommodation of considerably more anions. Therefore, through controlling the types of the solvated molecules, number of molecules in the solvated shell, and possible gas production, the blisters control the deformation below the elastic limit and are sufficient to avoid graphite exfoliation. Moreover, the volume change of the blisters with undamaged surfaces is reversible, and thus, it can play the role of increasing the specific capacity of the graphite cathode.

### 3.2 Elastic Property of Graphene Blister

**Figure 9:**
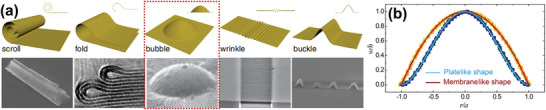
a) The schematics and corresponding electron microscope images illustrate various deformation modes. b) Normalized deflection profiles measured from two sets of 0.5‐µm‐radius bubbles (*w* and *r* represent weight (symbol *h*) and radius (symbol *a*) of the bubble, respectively). Reproduced with permission.^[^ ^41^ ^]^ Copyright 2019, American Physical Society.

Why do blisters exhibit such excellent elasticity without damaging the surface lattice? The explanation is that once a blister is produced, the number of graphite layers in the blister shell is often less than 10, and the physical properties of the blister shell, similar to those of multilayer graphene, differentiate it from bulk graphite, so we can refer to it as “graphene blister.”^[^ ^40^ ^]^ Furthermore, we observed that researchers in the nano‐mechanics field have begun studying the elastic mechanics of bent 2D materials, such as graphene, at the nanoscale. The multilayer 2D materials can bend in five ways: Scroll, fold, bubble, wrinkle, and buckle, as shown in **Figure** **9**a. Note that the words “blister” and “bubble” are temporarily not distinguished in the first half of this section, because the same factors were not considered simultaneously in different research fields. If a bubble with a multilayered shell structure appears on the surfaces of bulk 2D materials, the transition between plate‐like and member‐like behaviors of the bubble can be achieved by controlling the ratio of the bubble height and multilayered shell thickness,^[^ ^41^ ^]^ and the difference between the two behaviors is shown in Figure 9b. According to the literature, the thickness of the bubble (or blister) shell formed during graphite oxidation is approximately equal to the thickness of the eight graphite layers (≈2 nm), which leads to a greater height‐to‐thickness ratio. This type of bubbles belongs to the member‐like behavior, and the theoretical predictions of the membrane‐like behavior are in good agreement with the results of the electrochemical graphite oxidation experiment, because that the theoretical prediction is extremely similar to the microscopically observed bubble morphology in the literature. The relationship between the shell thickness and graphene bubble (or graphene blister) shape matches the theoretical predictions in the nano‐mechanics study well. When the strength of the interlayer interaction arises, the bending stiffness of multilayer graphene is effectively increased. Therefore, once the graphene bubbles are formed, they will possess excellent bending properties.

In recent years, Wang et al. studied the bending properties of van der Waals materials with multilayer structure and reported that the in‐plane Young's modulus and tensile strength of bulk layered materials (such as graphite) is generally orders of magnitude higher than their interlayer shear stiffness and strength. The extreme mechanical anisotropy may enable interlayer sliding in the layered materials.^[^ ^41^ ^]^ Graphene bubble formation can lead to interlayer slip in the shell. In addition, in the classical mechanics theory, the elastic modulus is proportional to the cube of the layer thickness. However, because the interlayer stretching and interlayer slip in graphene bubbles were considered by Wang et al., an additional coefficient related to the number of graphene layers, and the interlayer force was added to the theoretical formula. Compared with other 2D materials, graphene has a relatively small coefficient of ≈0.05–0.2. Therefore, the influence of the layer number in the graphene bubble shell cannot significantly affect the elastic modulus, indicating that the layer number in the shell can be maintained over a wide range, and it is considerably easier to form graphene bubbles induced by anion intercalation.

Graphene bubble formation is a manifestation of the strong adhesion between graphene and its substrate, and the strong adhesion enables the graphene bubbles to exhibit a series of unique properties.^[^ ^42^ ^]^ In the field of solid mechanics, numerous theoretical and experimental studies have been performed on the elastic strength of graphene bubbles and their adhesion energy to substrates. For example, in 2010, Levy et al. studied the magnetic field behavior in graphene nanobubbles affected by the strong stress.^[^ ^43^ ^]^ Moreover, the relationships between the adhesion mechanics, elastic mechanics, and the geometry of graphene bubbles have been theoretically studied in depth. For example, Koenig et al. studied the law of the change in the adhesion energy and shape of the graphene bubbles, wherein the gas is stored, on SiO_2_ substrate. The authors proposed that single‐layer graphene exhibited the strongest adhesion to the substrate, and the height of the single‐layer graphene bubble was larger.^[^ ^44^ ^]^ The nanoscale adhesions of graphene to substrates are higher than those measured in typical micromechanical structures, and are comparable to solid/liquid adhesion, which is attributable to the ability of graphene to conform to the topography of the smooth substrates, consequently facilitating its interaction with a substrate that is more like a liquid than a solid. Although the substrate surface is rough, the van der Waals forces across this contact area play an important role, which is greater than the adhesion of gold‐coated submicron beams by a factor of ≈5. Furthermore, Bunch et al. reviewed the reported results of the adhesion energy of graphene to the substrate.^[^ ^45^ ^]^ The authors claimed that the nano‐sized graphene materials were considerably affected by the surface forces, and the stiffness of the materials decreased with size, which exhibits various nonlinear mechanical relationships, such as a cubic relationship between bending stiffness and multilayer graphene thickness. When the graphene bubble is formed, its adhesion energy to the substrate can be expressed as

(2) $$\gamma =Ehw^{4}/16a^{4}$$

where *γ* is adhesion energy; *E*, stiffness; *a*, radius of the bubble; *h*, thickness of graphene bubble shell; *w*, height of graphene bubble.^[^ ^46^ ^]^ High adhesion allows a high flexibility of graphene bubbles. It can also be observed from this formula that graphene bubbles tend to form spherical bubbles with larger radii but lower heights because of the relatively weak interaction between the graphite layers, which is also lmost the same phenomenon observed in electrochemical graphite oxidation.

Whether it is a bubble or blister, it refers to the same research object, that is, the bulge on the graphite surface in different fields. However, the elastic properties of the graphene bubble when the bubble contains a liquid are different from those when it contains gas. Although most studies in the field of electrochemical oxidation of graphite have repeatedly mentioned the existence of gas in the bubbles when describing the bubble formation mechanism (during the de‐intercalation process, the overflow of gas causes the bubbles to become larger), and it was believed that the appearance of graphene bubbles was related to gas evolution;^[^ ^47^ ^]^ however, sufficient experimental evidence to demonstrate the existence of the gas in the bubble and the its escape mechanism remains lacking. Past analyses of this hypothesis have relied on the possibility of generating gas via solvent oxidation. Thus, determining whether the bubble contains gas or liquid requires further research. In 2018, Sanchez et al. studied the morphology of a graphene bubble containing liquid and its adhesion energy to the substrate.^[^ ^48^ ^]^ Although no consensus exists on whether the blisters are filled with gas or liquid, adhesion is a well‐established controlling parameter for blister formation as aforementioned. The authors reported that the subtle nature of the content inside the blister may render invalid the previously reported assumption that bubbles contain gas. Compared to the well‐established adhesion measurements, the direct application of this model resulted in unrealistically small adhesion values for graphene. Blister formation is energetically favorable only when the adhesion between the blister and substrate is sufficiently strong. The results of the elastic membrane model established by Sanchez et al. demonstrated that if the liquid volume (*V*∝*a* ^2^ *h*, *a* is the blister radius; *h* is the blister height) remains unchanged, the elastic energy decreases, and the interface energy increases with increasing bubble diameter *a*. For a liquid‐filled (water) blister, the mathematical relationship of the height/diameter ratio can be expressed as:

(3) $$\frac{h}{a}={\left(\phi \frac{\Gamma -\gamma_{w}\left(\cos\theta_{m}+\cos\theta_{s}\right)}{E_{2D}}\right)}^{1/4}$$

where *Γ* is the work of adhesion (or adhesion energy) of the membrane‐substrate interface; *γ*_w_ is the surface tension of water (≈0.072 J m^−2^); *θ*_s_ and *θ*_m_ are the water contact angles of the substrate and membrane, respectively; *h* and *a* represent the height and diameter of the blister, respectively; *E*_2D_ is the in‐plane elastic stiffness.^[^ ^48^ ^]^ The theoretical model showed that the weak shear limit (complete exfoliation of graphene owing to interlayer slip caused by the blister formation) predicts a larger aspect ratio *h*/*a* of the blister compared with that of a strong shear limit (it is challenging for the blister shell to withstand bending resulting in hard cracking), particularly for graphene (Poisson's ratio *ν* = 0.165).

**Figure 10:**
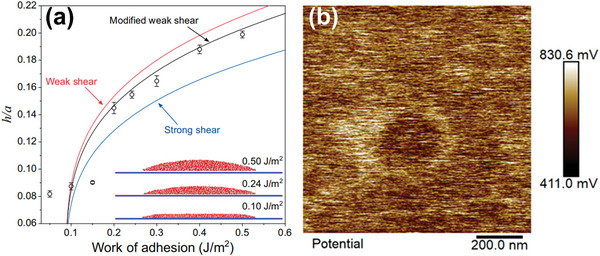
a) Modeling and MD simulations of water‐filled blisters. (Inset) The figure demonstrates how the shape of the blister changes for different values of the work of adhesion. Reproduced with permission.^[^ ^48^ ^]^ Copyright 2019, Akademisyen Kitabevi. b) The local surface potential characterization for contact potential difference (mV) value of an individual blister (measured from PF‐QNM (PeakForce‐Quantitative nanomechanical)). Reproduced with permission.^[^ ^49^ ^]^ Copyright 2018, American Chemical Society.

Further molecule dynamics (MD) simulation results (**Figure** **10**a) showed that for a larger adhesion energy, the aspect ratio *h*/*a* of the blisters was moderately large, and the blisters with excessive height might cause self‐rupture and surface damage, which is inconsistent with previous experimental results that the surface structures of graphene blisters caused by anion intercalation is maintained undamaged. However, in another case of the lower adhesion energy (<0.2 J m^−2^), the top of blister with low aspect ratio *h*/*a* was nearly flat. The adhesion energy of graphene on HOPG is 0.086 ± 0.016 J m^−2^, and thus, the top of the graphene blisters produced by the electrochemical graphite oxidation are predominantly nearly flat.^[^ ^49^ ^]^ This prediction is extremely similar to the microscopically observed blister morphology in the literature on the electrochemical oxidation of graphite in Section 3.1. In addition, according to the literature in the field of graphite oxidation, if the electrolyte solvent is water, the gas inside the blister is generated from the oxidation of carbon atoms, which is primarily based on the reaction formula C + 2H_2_O → CO_2_ + 4H^+^ + 4e^−^, indicating that 2 mol of water produces 1 mol of CO_2_. Then the volume of the blister increases by a factor greater than 100, which is inconsistent with the phenomenon observed via AFM. There are at least two reasons why it is contradictory: i) Only a significantly small quantity of gas in the blister, or ii) the gas is highly compressed owing to the high elastic stiffness of the graphene blister. However, no reported study is devoted to this topic. Based on the above analysis, the matter contained in the blister generated by the electrochemical oxidation of graphite is more likely to be liquid. Similarly, the graphene blister that appears on the graphite cathode surface in the DIB undergoing the same electrochemical process is also likely to be liquid, that is, intercalated solvated anions. We can now distinguish between the word “blister” and “bubble,” which contain liquid and gas, respectively.

If no gas exists in the blister, then another important phenomenon should be considered. Research teams varying with countries or regions have reported the same phenomenon: After first charge/discharge cycle, graphene blisters grow larger under lower voltage, but decrease in size under higher voltage, which does not appear to be a logical electrochemical behavior. Because the solvated anions are more likely to intercalate to form graphene blisters under higher voltage, consequently leading to larger blisters, whereas, the experimental phenomenon is completely opposite. There were no ready‐made logical connections in the literature linking these contradicting results from those different fields; therefore, we provide a possible explanation: The blister serves the accommodation of reactants. Under higher voltage, solvated anions intercalate into the surrounding lattice connected to the inner blister, and then the loss of ions and solvent decreases the blister sizes; under lower voltage, the ions in the lattice are de‐intercalated and returned to the blister, so the surface structure is not damaged, and the electrochemical process is reasonable. Additionally, the solvated anions are de‐intercalated and returned into the graphene blisters rather than the electrolyte near the cathode surface possibly owing to the Marengoni effect caused by the high elastic stiffness of the graphene blisters; this has not been reported in the literature on micro‐ and nano‐sized liquid blisters or bubbles.^[^ ^50^ ^]^ Tripathi et al. reported a logical plausibility to this explanation.^[^ ^49^ ^]^ They characterized the local potential distribution on a single blister surface, as shown in Figure 10b. The results demonstrated that a significant potential difference exists between the blister edge and blister center, and the surface potential at the blister center decreased with increasing graphene blister height, consequently reducing the local work function (eV). According to the basic principle of electrode potential in electrochemistry, a reduction in the electrical work required for external electrons to reach the surface of the blister decreases the external potential and subsequently reduces the electrode potential. The surface of the graphene blister with lower electrode potential is not equivalent to the equipotential surface of bulk graphite. The blister cavity is equivalent to a miniature electrolytic cell, which creates the optimal electrochemical condition for the anion backflow into the blister.

The above analysis suggests that a core issue in increasing the specific capacity of DIBs is the controllable formation and reversible deformation of graphene blisters. The intercalation energy is related to the operation voltage of the DIB, however, previous research predominantly considered the simplest condition, that is, the intercalation of anions into the graphite interlayers.^[^ ^51^ ^]^ Therefore, for graphene blister formation, the elastic stiffness of graphene blisters also requires to be considered when calculating the theoretical operation voltage from the difference between the intercalation energy and desolvation energy. Moreover, as mentioned above, blistering caused by anion intercalation always occurs in the vicinity of the graphite subsurface at the beginning and subsequently proceeds to the deeper layers, which is explained in this section: The shell thickness of graphene blisters is positively related to the elastic stiffness of the multilayer graphene, so the maximum elastic energy and number of shell layers of graphene are determined by the interlayer force of graphite and intercalation ability of anions, which facilitates the appearance of blisters in the subsurface below 5–12 surface layers. After the formation of graphene blisters in the subsurface layer, the deeper graphene blisters continue to be generated. It can be inferred from the conclusions that a high interlayer force requires to be overcome during the 1st intercalation of anions to form graphene blisters, and in the subsequent intercalation/deintercalation cycles, only relatively low elastic moduli of graphene blisters require to be overcome. The DIB will predictably have a high charge/discharge voltage plateau in the first cycle, and will gradually decrease in the subsequent cycles, however, the specific capacity exhibits a significantly opposite trend to that of the voltage plateau, and the specific capacity gradually increases owing to blister formation and its excellent anion holding capacity. When the number of graphene blisters reaches a threshold value, the voltage platform and specific capacity tends to stabilize, which is almost a normal phenomenon in the long‐term stability tests of DIBs.^[^ ^52^ ^]^

### 3.3 Relationship between Graphene Blister and Graphite Grain Boundaries

In 2018, Sinitsyna et al. observed an important phenomenon while preparing graphite oxide via the Hummers method. The blisters on the graphite surface caused by the intercalation of SO_4_^2−^ anions were generated along the same line‐shaped trajectory as the graphite grain boundaries.^[^ ^53^ ^]^ The graphitic structure of the top layer on the blister remained intact, and this result was consistent with those reported by McCreery et al. in 1992. Furthermore, in some previous studies, the researchers claimed that pre‐existing defects on the graphite surface contribute to blister formation by serving as stable ion channels and supporting the reversible transport of ions in the blisters.^[^ ^33^, ^34^ ^]^ Subsequent reports have indicated that this defect actually refers to the GBs. Graphite GBs, also known as “superlattices,” are produced by the contact of two graphite lattices at different angles in the same plane.^[^ ^54^ ^]^ Then, the entry mechanism of the solvated anions into the lattice through the graphite GBs to form graphene blisters is a topic that requires further exploration, and it can help to realize the controllable formation of graphene blisters.

**Figure 11:**
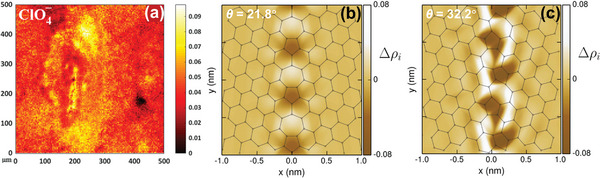
a) Normalized ToF‐SIMS images (500 µm × 500 µm) obtained in negative‐ion mode from HOPG after treatment with 2 m HClO_4_ solution of ClO_4_^−^ ion signals. Reproduced with permission.^[^ ^55^ ^]^ Copyright 2019, American Chemical Society. b,c) The calculated charge distribution along the grain boundaries for the *θ* = 21.8° and *θ* = 32.2° GB, respectively (*θ*, misorientation angle) (the symbol ∆*ρ_i_* means excess charge (in units of electrons per carbon atom)). Reproduced with permission.^[^ ^57^ ^]^ Copyright 2016, IOP Publishing Ltd.

Although this phenomenon has long been discovered, it was only in recent years that advanced characterization instruments were used to further analyze the formation process of graphene blisters grown on GBs and the specie compositions in the blisters. In 2019, Rosa et al. used time‐of‐flight secondary ion mass spectrometry (ToF‐SIMS) to analyze the chemical element distribution and change in chemical compositions on the HOPG surface before and after electrochemical oxidation of HOPG in the electrolyte.^[^ ^55^ ^]^ When HOPG was electrochemically treated in perchloric or sulfuric acid, the intensities of the characteristic ionic peaks of graphite, such as C^2−^, C^3−^, C^4−^, C^5−^, and C^6−^, were significantly reduced, and the mass spectrum in the negative‐ion mode showed a stronger oxygen ion peak compared with that of the pristine graphite surface. These results suggested that the carbon atoms in the topmost lattice of HOPG were oxidized by the aqueous electrolyte to form graphitic oxides. In addition, a few chemicals, including chloride Cl^−^, perchlorate ion ClO_4_^−^, sulphite ion SO_3_^−^, bisulfate ion HSO_4_^−^, and solvated ClO_4_^−^, related to the anion intercalation process were also detected. These ion signals were stronger at fractures on the graphite surface (**Figure** **11**a). Two important observations can be made here: i) The electrochemical intercalation of anions is accompanied by the partial oxidation of graphite (if the solvent is water), which does not conflict with Murray's finding detected using EDX and auger electron spectroscopy that graphite oxide on the top surface of the inner blister was covered by the 5–12 undamaged outer layers;^[^ ^35^ ^]^ ii) Solvated ions are involved in the intercalation reaction. Moreover, through analyzing the signal distributions of the intercalation of various chemical species, the authors concluded that graphitic defects (i.e., GBs) were the preferred initiation sites for the electrochemical anion intercalation process. It has also been proposed in other studies that electrochemical oxidation at the edge sites and GBs subsequently leads to the expansion of the graphite layers, thereby facilitating anion intercalation within the graphitic layers in both ionic liquid and aqueous solution.^[^ ^56^ ^]^

It is already known that the initial intercalation of anions at graphite GBs is required for blistering and subsequent reactions. Combined with the literature in the field of condensed matter physics studying the electronic structure of graphite or graphene GBs, we explain the preferential intercalation of anion into GBs. The graphite GB is a 1D line‐shaped structure comprising two graphite lattices with different angles. Along the GB, a periodic structure of pentagon‐heptagon chain has been observed.^[^ ^57^, ^58^ ^]^ This unique periodic superstructure (or superlattice) affects the surface charge density distribution of graphite. Simulation results demonstrated that positive charges were concentrated on the heptagon, whereas negative charges were concentrated on the pentagon, consequently resulting in a dipole moment at the GBs (Figure 11b,c). This effect of GBs on the electronic structure, decays rapidly within a short distance of 2 nm. Therefore, the GBs with dipole moments are more likely to adsorb anions than other regions of the graphite basal plane.

**Figure 12:**
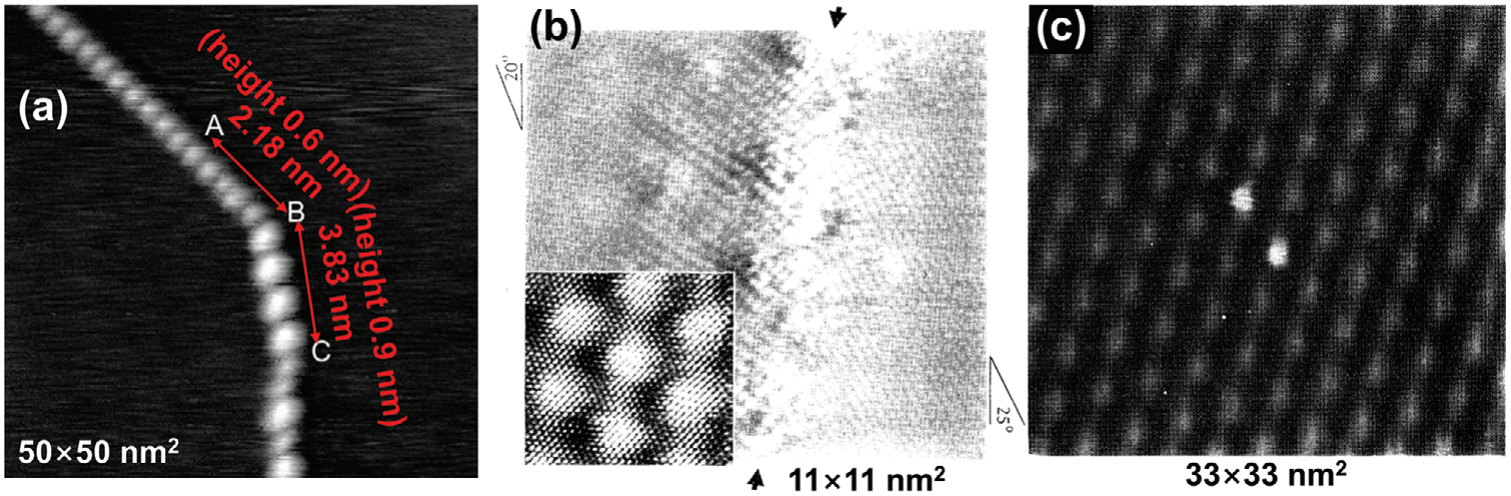
a) STM image of 1D superlattice on a flat HOPG surface with two periodicities. b) A STM image taken near a boundary of a giant lattice with a lattice constant of 2.8 nm. The inset shows an overview (7 × 7 nm^2^) of this giant lattice taken at the left‐hand side of the boundary. c) A STM image showing two cobalt particles on the top site of the giant lattice. a) Reproduced with permission.^[^ ^59^ ^]^ Copyright 2007, IOP Publishing. b,c) Reproduced with permission.^[^ ^60^ ^]^ Copyrigtht 1993, American Physical Society.

There is sufficient evidence to support this explanation. Cervenka and Flipse studied the GBs on the surface of HOPG, and their results demonstrated that the 1D superstructure has a higher charge density compared with that of the normal graphite surface, which makes it easier for GBs to adsorb atoms and molecules.^[^ ^59^ ^]^ Using AFM, which is operated in contact or tapping modes, the authors have not observed GBs on HOPG, and proposed that the GBs were not induced by topography but have a purely electronic origin. Moreover, in STM images through measuring the tunneling current between the tip and the sample, the GB exhibited a higher charge density than the normal graphite surface, and the highest point of the charge density on the GB has periodicity (**Figure** **12**a). The authors analyzed a simple physical model based on electron wave interference and provided the reason for this phenomenon: the GB periodicity arises from the 1D graphene Moiré pattern produced between two rotated electron waves, and a threefold symmetric electron scattering from the GB. Moreover, Xhie et al. observed 1D GBs using STM and recorded the results of adsorption of cobalt atoms on the graphite GBs (Figure 12b,c).^[^ ^60^ ^]^ These results showed the highest charge density at the vertices of the GB.

Furthermore, anion adsorption is followed by the desolvation and intercalation processes. Schnyder et al. studied the relationship between anion intercalation and friction on HOPG and provided experimental evidence to support the presence of desolvation.^[^ ^61^ ^]^ They used X‐ray photoelectron spectroscopy to analyze the element distribution of the electrochemical double layer constructed via anion adsorption and the element distribution on the graphite surface after intercalation. They experimentally demonstrated that the number of water molecules involved in solvation shell decreased significantly after the intercalation of ClO_4_^−^ ions. These results indicate that although the intercalation of solvated anions into the GBs leads to blister formation, the desolvation process remains. Yoshio et al. studied graphite cathodes, and reported that GBs could block the diffusion of macromolecules, while allowing smaller desolvated ions to intercalate.^[^ ^62^ ^]^ In terms of studying the mechanism of ion intercalation, although a few researchers have studied the intercalation behavior of anions on normal graphite composed of hexagons, the mechanism of anions intercalation at the GBs has rarely been studied.^[^ ^63^ ^]^ However, studies on the application of graphite GBs in the fields of energy storage devices or energy conversion other than DIBs have been performed. For example, the graphite GBs with unique electronic and structural properties were used as the support sites of single atoms in single‐atom catalysts for enhancing the adsorption of active intermediate.^[^ ^64^ ^]^ Additionally, the layered material was expanded via intercalation at the GBs, thereby increasing the specific surface area of the electrode materials in aqueous flow batteries.^[^ ^65^ ^]^ Therefore, it is particularly important to study the anion intercalation on graphite GBs and the subsequent reversible behavior of graphene blisters. A few useful in situ characterization instruments, such as ToF‐SIMS, can be used, which has been mentioned in the reported review of DIBs.^[^ ^66^ ^]^

## 4 Correlation between the Blistering and the Battery Performance

### 4.1 The Performance Enhanced by Blistering on Graphite Cathode in Dual‐Ion Batteries

How the blistering phenomenon affects the battery performance and the internal cause also need to be further explored. Very little published articles are available focusing on the surface blisters of graphite cathodes in the field of DIBs. Nevertheless, some valuable information and conclusions can still be obtained according to the published results that related to the surface blisters on graphite anodes.

Inaba et al. have noticed the blistering phenomenon on HOPG surface in EC‐based electrolytes and studied its effect on electrode performances in ref. [67]. When using 1 m LiClO_4_ dissolved in EC‐based solvent as the electrolyte, a special blister structure would appear on the graphite surface during the initial charging. The authors suggested that the blistering was due to the intercalation of solvated lithium ions and the accumulation of their decomposition products, that is, the formation of SEI. This process was crucial for the formation of stable graphite/electrolyte interface. In 1999, Inaba's research group continued to study the influences of ion intercalation in trifluoropropylene carbonate (TFPC) solvent on the surface morphology of HOPG.^[^ ^68^ ^]^ The experimental results verify the previous conclusion that the formation of SEI (contributing to the blister structure) on the graphite was triggered by the intercalation of Li(TFPC)*_n_*, and the SEI was formed rapidly at step edges before graphite layers began to exfoliate. Therefore, the stability of the graphite against solvent co‐intercalation is one of the key factors influencing the stability of the SEI formation. However, the “blister” obtained in TFPC was quite distinct from the blister on the graphite surface in EC‐based electrolytes. The STM image showed that the top of the blisters obtained in the EC‐based electrolyte exhibited the same bright spots at intervals of ≈0.25 nm as the graphite basal surface, indicating that the blister was an interior structure formed under the surface.^[^ ^67^ ^]^ In contrast, the decomposition products of Li(TFPC)*_n_* were exposed on the surface rather than the subsurface. Meanwhile, compared with EC‐based electrolytes, TFPC‐based electrolytes exhibited larger initial irreversible discharge capacity (120 mAh g^−1^ for EC/DEC; 335 mAh g^−1^ for TFPC), as well as smaller reversible discharge capacity (340 mAh g^−1^ for EC/DEC; 275 mAh g^−1^ for TFPC). Therefore, the subsurface blister with an intact graphite structure was more beneficial to improve the reversible capacity of the batteries.

**Figure 13:**
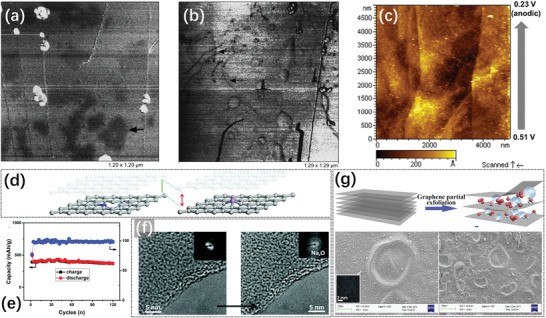
a) AFM phase images of the HOPG surface in the electrolyte containing VC or b) in the blank EC‐based electrolyte. Reproduced with permission.^[^ ^69^ ^]^ Copyright 2002, Elsevier B.V. c) The in situ SPM images of the HOPG basal plane at anodic 0.51–0.23 V in 1 mol L^−1^ LiFSA in PC:tetraglyme. Reproduced with permission.^[^ ^70^ ^]^ Copyright 2016, Springer. d) Schemes of in‐plane “hole” and “protrusion” structures. e) The cycling performance of graphene with protrusions at 25 mA g^−1^. f) HRTEM images of the surface structural evolution of graphene with protrusions during the discharging process. Reproduced with permission.^[^ ^71^ ^]^ Copyright 2017, Royal Society of Chemistry. g) Graphical mechanism for electrochemically partial exfoliation of graphene sheets from graphite foil, and SEM images of the electrodes after galvanostatic charge/discharge for 1000 cycles. Reproduced with permission.^[^ ^21b^ ^]^ Copyright 2014, Elsevier B.V.

The above conclusions were also verified by the results published by Matsuoka et al. in 2002.^[^ ^69^ ^]^ They studied the decomposition precipitation, swelling at step edges, and blistering that occurred on HOPG surface in the electrolyte of LiPF_6_ EC:EMC when lithium was intercalated. The authors believed that the swelling at step edges is the SEI played an important role in the intercalation/de‐intercalation of lithium ions. Subsequently, a layer of decomposition products was covered on the basal plane of HOPG after adding vinylene carbonate (VC) additive to the electrolyte, which could cause the isolating of the electrolyte and graphite, while maintaining the swelling at step edges and blistering. The phase image of AFM showed that there was a phase contrast between the “blister” and the basal plane in the electrolyte containing VC. In contrast, there was no significant phase contrast between the blister area and the basal plane in the EC‐based electrolyte (**Figure** **13**a,b), and the graphitic structure at the top of the blister was intact. The results showed that the SEI formed by VC was composed of the decomposition product layer on the graphite surface, while the blister existed between the graphitic interlayer below the surface. The latter was more conducive to the intercalation and de‐intercalation of ions. Moreover, in 2016, Song et al. also found that the co‐intercalation of PC‐solvated lithium ions at low potential would cause the formation of blisters (Figure 13c), and the decomposition products of PC solvated molecules in the blisters constructed the SEI, which helped prevent further intercalation of other solvated molecules and improved the stability of the electrode structure.^[^ ^70^ ^]^ Stable blisters and internal SEI would also be formed in the electrolyte of LiCF_3_SO_3_ (LiOTf) in PC:tetraglyme (20:1 *v*/*v*), and the anion of OTf^−^ could perform stable reversible intercalation/de‐intercalation process in graphite. The discharge capacity remained ≈300 mAh g^−1^, and the discharge curves coincided well. Indeed, these blisters mentioned above all showed the characteristics of blistering along the step edges of the basal plane, consisting with the previous conclusion that the blister was preferentially formed at the defects on basal plane.

Furthermore, Yang et al. in 2017 discussed the effect of “protrusions” and “holes” in graphene electrodes on the sodium storage performances.^[^ ^71^ ^]^ The authors designed phosphorus‐doped and nitrogen‐doped graphene anodes to introduce “protrusion” (similar to blister) and “hole” structures (Figure 13d), respectively, and systematically investigated the “protrusion” and “hole” effects in graphene toward sodium‐ion storage, which further unveiled the enhanced electrochemical performance of protrusions at the atomic level. Compared to the graphene with hole, the graphene with protrusions showed superior electrochemical performances, exhibiting an ultrahigh capacity of 374 mAh g^−1^ at 25 mAg^−1^ even after 120 cycles (Figure 13e), as well as an ultrafast energy storage toward sodium (210 mAh g^−1^ at a very high current rate of 500 mA g^−1^). DFT calculation results showed that the enlarged (0002) spacing of graphene with protrusion could bring in more Na^+^ storing sites, which would greatly facilitate the mobility of both electrons and Na^+^ ions during the sodiation process (Figure 13f), thus leading to excellent rate capability of graphene electrodes. Therefore, the authors concluded that introducing protrusions (or blisters) into the graphene‐based electrodes would be an effective way to storage more Na^+^ and to further improve the capacity of the batteries.

The blistering on graphite can also enhance the electrochemical performances of other energy storage devices, for instance, the supercapacitors. Liu's group prepared partially exfoliated graphene through anion intercalation into multilayer graphene in phosphate buffer solution (pH = 6.68) containing 1 m KNO_3_.^[^ ^21b^ ^]^ Subsequently, they conducted the electropolymerization of obtained partially exfoliated graphene in the solution of 0.2 m pyrrole containing 0.05 m 2‐naphthalene sulfonic acid to prepare the partially exfoliated graphene decorated with polypyrrole (PPy). It was found that the new graphene electrode was compounded with 2‐naphthalene sulfonate that provide continuously anion SO_3_^2−^.^[^ ^21b^ ^]^ The self‐stand film exhibited good elasticity and made it possible to form blisters after swelling of the polymer film during charge/discharge process (Figure 13g). The author used a tweezer to puncture the blister and the electrolyte flowed out, therefore, the phenomenon indicated that the electrolyte could reach the inner surfaces of blisters and both sides of the blisters could be used for charge storage. After 1000 charge/discharge cycles, the blisters could still be existed and the capacitance of the film lost only 9% compared with its initial capacitance (from 257 to 234 F g^−1^). Specifically, the specific capacitance only showed a sharp decline in the initial 100 cycles and then displayed insignificant changes in the following 900 cycles (97% is maintained from 242 to 234 F g^−1^), demonstrating the improved structural stability of PPy anchored partially exfoliated graphene electrodes. It is worth noting that the other sample prepared by the authors did not exhibit the blister structure. The difference between the two samples was that they contain different matters that continuously donated anions. As a result, the absence of the blister structure led to a poor rate performance of the electrode (lost 18% of initial capacitance after 1000 cycles).

In conclusion, the graphene blisters on the graphite surface exert a great impact on the battery performance. That is, the ability of the electrode to accommodate ions can be enhanced, and a stable electrode/electrolyte interface by forming the SEI in the inside can be obtained through forming the blisters. However, currently little information is available concerning the role of blisters in the battery field. In view of this, the effect of the blisters on battery performance and its microscopic mechanism still need to be further studied.

### 4.2 Missing Reversible Blister Model in Dual‐Ion Battery Mechanism

In the field of DIBs, a few unique experimental phenomena related to blister formation have been neglected by researchers. These abnormal phenomena can easily relate to graphene blisters by reviewing the literature in several research fields including DIBs, electrowetting, and electrochemical oxidation of graphite, and considering the previous discussions.

**Figure 14:**
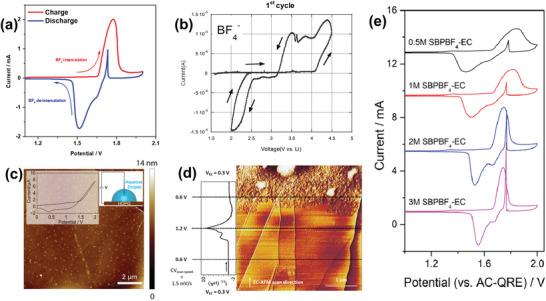
a) CV curve of graphite cathode in the electrolyte of 1.5 m SBPBF_4_ (spiro‐(1,10)‐bipyrrolidinium tetrafluoroborate) in EC. Reproduced with permission.^[^ ^72^ ^]^ Copyright 2019, American Chemical Society. b) First CV curve in SWNT/Li cell using the electrolyte of EC:DMC‐LiBF4. Reproduced with permission.^[^ ^74^ ^]^ Copyright 2006, Springer. c) 10 µm × 10 µm AFM images of HOPG after removal of a droplet of a 1 mm NaClO_4_ solution that had resided on the surface with the voltage swept from 0 to 2 V for 30 cycles (1 V s^−1^). Reproduced with permission.^[^ ^76^ ^]^ Copyright 2016, American Chemical Society. d) In situ and real‐time EC‐AFM acquisition: synchronized with the CV sweep. Reproduced with permission.^[^ ^77^ ^]^ Copyright 2019, Elsevier B.V. e) The CV curves of graphite cathode using the electrolytes of SBPBF_4_ in EC with different concentrations. Reproduced with permission.^[^ ^72^ ^]^ Copyright 2019, American Chemical Society.

In 2019, Wang et al. explored the abnormal behavior of BF_4_^−^ anions on graphite surfaces when the cyclic voltammetry (CV) curves of the intercalation/de‐intercalation of the anions in EC solvent were measured. The authors observed an abnormal oxidation current peak in the discharge segment of the CV curve (de‐intercalation process) and referred to it as “countercurrent,”^[^ ^72^ ^]^ as shown in **Figure** **14**a. The CV‐quartz crystal microbalance technique and in situ electrochemical dilatometric method were performed to analyze this abnormal phenomenon. The results demonstrated that the appearance of the countercurrent was related to whether the EC solvent entered the graphite lattice. If the carbon layer‐coated spheroidal graphite was used, the EC solvent molecules were completely removed at the interface, and then the countercurrent disappeared. Moreover, the expansion curves of the electrodes during the discharge deintercalation process showed a significant electrode expansion peak near 1.7 V, which corresponds to the onset potential of the countercurrent. The authors had specifically reported that the EC solvent molecules undergo delayed de‐intercalation after intercalation in the graphite lattice.^[^ ^14^ ^]^ Therefore, a preliminary explanation was provided by the authors: the EC solvent inevitably hinders BF_4_^−^ intercalation owing to the strong interaction between BF_4_^−^ and EC, leading to the partial intercalation of BF_4_^−^, and the rest of anions remain outside the graphite. Subsequently, the intercalated BF_4_^−^ re‐solvates during de‐intercalation. If the re‐solvation occurs in the graphite lattices, the entry of BF_4_^−^ immobilized outside the graphite electrode into the graphite lattice causes a backflow process, resulting in the abnormal counter oxidation current, because the BF_4_^−^ immobilized outside the graphite electrode also requires to be re‐solvated by EC solvent molecules before appearing as the ionic current of de‐intercalated BF_4_^−^. After reviewing the literature, we observed that the countercurrent in the CV curve likely occurs when the anions and solvents that are easier to intercalate into the graphite lattice are used. For example, in the following work, Wang et al. used GBL solvent to replace the EC solvent. The co‐intercalation of BF_4_^−^ and the EC solvent was suppressed owing to the weak interaction between GBL and BF_4_^−^, and thus, the de‐solvation of internal BF_4_^−^ in the graphite lattice was reduced, resulting in the disappearance of the phenomenon of countercurrent.^[^ ^73^ ^]^ Therefore, the smaller BF_4_^−^ anion sizes and its stronger interaction with the solvent are the factors that lead to the countercurrent phenomenon. Moreover, the authors also observed that the strong interaction between Li^+^ and BF_4_^−^ could regulate the BF_4_^−^ intercalation properties if the main salt of SBPBF_4_ was replaced by LiBF_4_. Although a countercurrent peak was also observed in this case, a higher cut‐off voltage was required to induce this phenomenon, which was more evident in the experiments conducted by Yazami et al. who also thought it was an “anomaly.”^[^ ^74^ ^]^ When they used the LiBF_4_ electrolyte in EC‐DMC to study the anion intercalation behavior of anions into single‐walled carbon nanotubes, they observed that a wide anomalous anodic reverse current appeared in the initial de‐intercalation segment of the CV curve (Figure 14b). However, the authors did not provide an explanation. Although the authors did not mention graphene blisters in their paper, we have noticed that the graphite surface blister was a typical phenomenon observed in EC solvent‐based electrolytes.^[^ ^75^ ^]^

The results of the electrowetting experiments on the HOPG surface conducted by Unwin et al. appear to be closer to the answer.^[^ ^76^ ^]^ The authors investigated the relationship between anion intercalation and electrowetting by observing the wetting behavior of the droplets of 5 µL NaClO_4_ aqueous solution on HOPG under different voltages. A two‐electrode configuration was applied to the assembly of the droplet cell, wherein the HOPG sample was employed as the working electrode and an AgCl‐coated Ag wire into the droplet served as a combined counter/reference electrode. The experimental results demonstrated that the highest quality HOPG samples with lower step density (with monolayer step) were more likely to lead to ion intercalation and de‐intercalation, consequently resulting in significant electrowetting. In contrast, the HOPG samples with a considerably higher step edge density (with multilayer step) exhibited considerably less electrowetting. It seems illogical that more edge planes cannot increase amount of intercalation sites. Furthermore, although the HOPG samples with higher multilayer step density underwent potential scans under the same test condition, no significant intercalation and de‐intercalation peaks appeared, indicating that the voltage was not the only factor affecting the intercalation/de‐intercalation of anions. However, the authors simply attributed this inability to the electrochemical intercalation of anions to the strong binding force imposed by the high density of multilayer step edges. Additionally, the authors conducted a series of experiments for analyzing the abnormal phenomenon, including the operando characterizations of contact angle (CA) and relative contact diameter (RCD) during the CV curve measurements. The droplet was gradually spreading with increasing voltage. The results of the CA and RCD tests showed that the RCD increased by 8% when the voltage increased from 0 to 1.13 V, and the CA decreased from 51° to 38°. When the voltage reverted to 0 V, the RCD curve, which should be immediately decreased, exhibited an abnormal increase in during the de‐intercalation process. A similar phenomenon was also observed in the CA curve, which was not synchronized with the reverse voltage. The authors referred to this phenomenon as “hysteresis” that was repeatable and reversible, and they proposed that hysteresis occurred because of the change in potential under a certain scan rate was faster than the wetting rate. Notably, the AFM images of the wetting area showed the formation of dense blisters on the HOPG surface after 30 intercalation/de‐intercalation cycles (Figure 14c). The authors did not provide an explanation and only mentioned the presence of point defects (i.e., blisters) on the basal plane. However, ClO_4_^−^ used in this experiment is an anion that easily intercalates and forms graphene blisters; therefore, it is evident to us that a strong link exists between this abnormal phenomenon of anion intercalation and the physical properties of graphene blister.

As aforementioned, blister formation induced by the electrochemical oxidation of graphite has been fully demonstrated. The relationship between the blister size and voltage showed an anomaly that does not conform to general electrochemical phenomenon: the blister is small when the voltage is high, and the blister is large when the voltage is low, which is related to the anomalies reported in the studies mentioned in this section. The discovery of the abnormal phenomenon of the blister came from different countries and regions as well as different laboratories, and this simultaneous appearance is not a simple coincidence. These anomalies all have one thing in common: They are all reversible and occur when the voltage shifts to a lower value, that is, during the de‐intercalation of anions.

Yivlialin et al. provided sufficient experimental evidence that blister formation is more favorable during the deintercalation process using advanced instruments, such as EC‐AFM (Figure 14d).^[^ ^77^ ^]^ The blister significantly appeared near the onset potential of the cathodic current peak. However, although the blisters were formed on the graphite surface, no countercurrent was observed in the CV curve, because the authors used SO_4_^2−^ or HSO_4_^−^ anions rather than the easily intercalated anions, which does not conflict with the facts that the countercurrent was easily induced by BF_4_^−^ and ClO_4_^−^. Therefore, it can be inferred that the appearance of the countercurrent indicates blister formation, but the countercurrent does not necessarily appear after the blister is formed. Moreover, Wang et al. also reported that the countercurrent peak position did not change with the change in electrolyte concentration (Figure 14e), which does not conform to the Nernst equation in electrochemical kinetics, indicating that the backflow is caused by a pure physical process. Thus, the largening of the graphene blisters during de‐intercalation process is used to explain the anomalies in DIBs and electrowetting, we can effectively conclude the following from all the contents in this review: During intercalation, the anions preferentially enter the lattice through the graphite GBs, resulting in graphene blister formation (5–12 graphene layers in the shell) with high elastic stiffness. In the process of reducing voltage, the blister absorbs the solvated anions de‐intercalated out of the lattice connected to it, which enlarges the blister, and the high blister elasticity causes the physical backflow phenomenon (we predict that this is due to the Marengoni effect). This delayed anion storage behavior causes the hysteresis behavior in CA and RCD curves in electrowetting. For convenience, we can refer to this behavior of graphene blister with predicted properties as the “blister model”, which is reversibly expanded and can repeatedly accommodate solvated anions.

**Figure 15:**
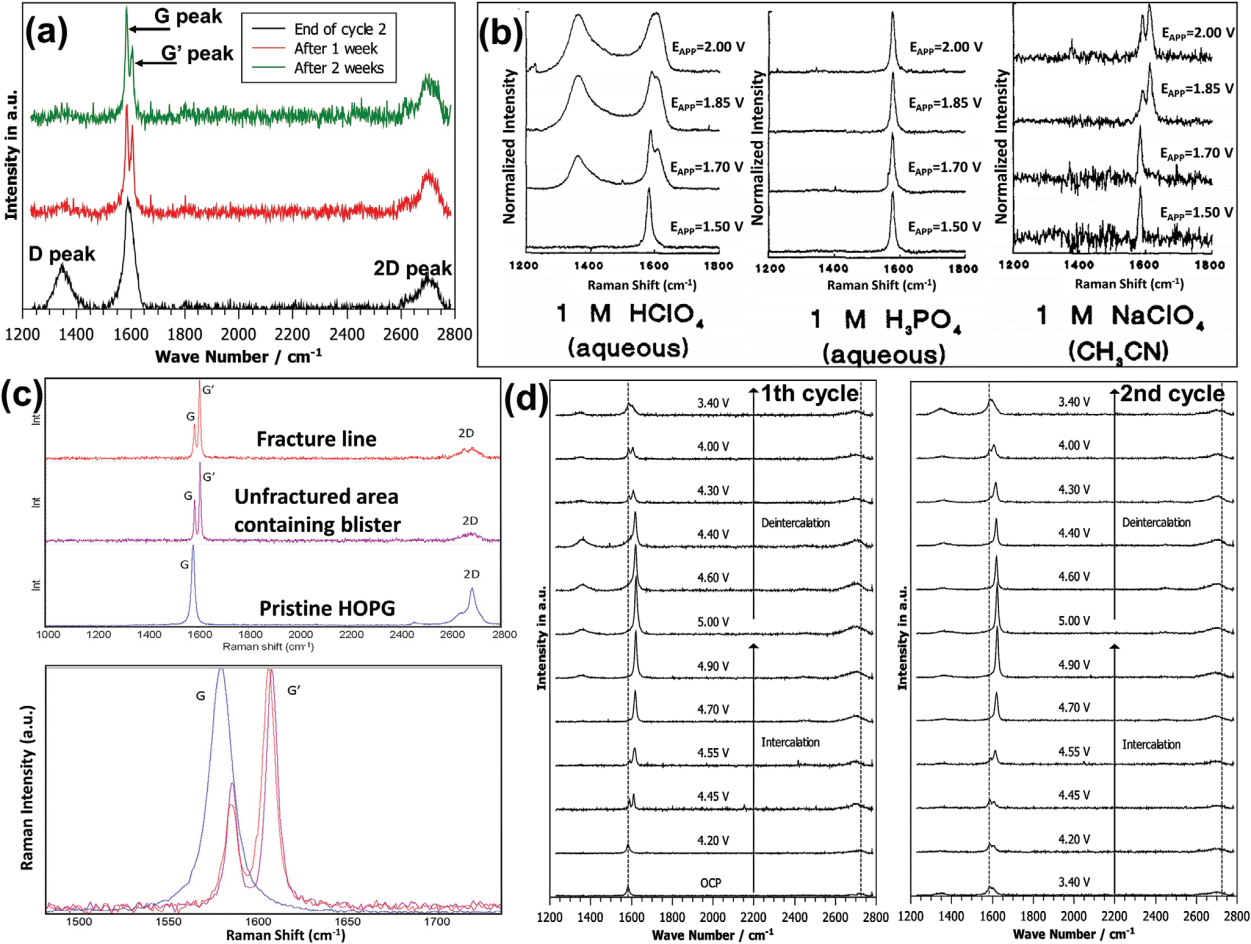
a) Normalized Raman spectra obtained at the end of the second cycle of the in situ Raman measurement and after 1 and 2 weeks of storage at room temperature. Reproduced with permission.^[^ ^78^ ^]^ Copyright 2016, Elsevier B.V. b) Raman spectra of HOPG after potential steps lasting 2 min from 0.5 V to four higher potentials in 1 m HClO_4_, 1 m H_3_PO_4_, and 1 m NaClO_4_/CH_3_CN. Baseline noise In the CH3CN case was due to subtraction of solvent bands. Reproduced with permission.^[^ ^33^ ^]^ Copyright 1992, American Chemical Society. c) Raman spectra during the CV after reaching the III intercalation stage, and their enlarged part of G‐G′ doublet peaks. Reproduced with permission.^[^ ^39^ ^]^ Copyright 2017, American Chemical Society. d) Raman spectra obtained during the first cycle and the second cycle. Reproduced with permission.^[^ ^78^ ^]^ Copyright 2016, Elsevier B.V.

Another inexplicable anomaly discovered by Balabajew et al. in their study on DIBs can also be explained by the “blister model”. When they studied the behavior of the storage anions of graphite foam in the electrolyte of LiTFSI in Pyr_1,4_TFSI, they observed that graphite defects caused by the intercalation/de‐intercalation reaction can self‐recover after standing.^[^ ^78^ ^]^ Raman spectroscopy was used to characterize this self‐recovery process, as shown in **Figure** **15**a. After two cycles, the battery was left to stand for 1–2 weeks, and the Raman spectroscopy results of the graphite electrode showed that the D peak representing disorder or defects disappeared, whereas the G peak representing the graphite lattice split into two parts (G–G″). The G″ peak begins to appear due to the increase of graphite layer spacing induced by anions intercalation. If the battery was recharged, the anions required to overcome the same interlayer force of graphite as that in the first cycle. The authors explained that the movement of the graphene layers causes defects to repair themselves. The appearance of the D peak and splitting of the G peak caused by anion intercalation have been discovered by McCreery et al. in 1992.^[^ ^33^ ^]^ As shown in Figure 15b, anion intercalation could destroy the lattice structure of graphite in 1 m HClO_4_ aqueous solution, leading to the appearance of the D peak, and the intensity of the D peak increased with increasing the number of intercalated anions. The G peak was not split and no D peak was observed in the absence of anion intercalation (using PO_4_^3−^). When the lattice was not damaged by anion intercalation in the electrolyte of NaClO_4_ in acetonitrile, only G peak splitting occurred. Thus, the G peak can represent the degree of structural perfection of blister shell, while the G′ peak symbolises anion intercalation and possible formation of the blister. These results indicate that graphene blisters should be generated in the experiments performed by Balabajew, and that such blisters are reversible, and can revert to graphite with strong interlayer forces. This phenomenon that graphene blisters can be recovered has only recently been strongly corroborated by the experiments conducted by Xu's group.^[^ ^79^ ^]^ They reported that the solution‐enclosing blister could be reversibly modulated by the solution osmotic pressure.

Referring to the study of Yivlialin et al. in 2017 investigating the relationship between graphene blisters and Raman spectra.^[^ ^39^ ^]^ When the anions were intercalated into the graphite and stage‐III was reached, thin lines and blisters appeared on the graphite surface. The thin line represents the fracture, and numerous small blisters are present around it, so these so‐called fracture lines are likely to be lines composed of several tiny blisters and cracks generated on the graphite GBs, which does not conflict with the conclusions in Section 3.3. The authors tested the Raman spectra of these lines, as shown in Figure 15c, and the red line was the result of the fracture line (it can be thought of as a blister line with considerably fine cracks), and the purple line was the result of the intact graphene blister surface, and the blue line was the spectrum of the pristine HOPG surface. The results demonstrated that the appearance of graphene blisters with an intact lattice structure did not cause D peak, and the G′ peak (arising from the splitting of G peak and located at a higher wave number) caused by anion intercalation shifted to a lower wave number after the cracking of the graphite surface, which is almost identical to the results of Raman spectra observed by Balabajew (Figure 15d). Moreover, Balabajew reported that the shift of the G′ peak to the lower wavenumber was accompanied by the appearance of the D peak, and this phenomenon appeared during the de‐intercalation process in the first cycle. In the second cycle, the D peak was more distinct at a low voltage, which reminds us of the abnormal electrochemical phenomenon of graphene blisters. Combined with the above conclusions, it can be inferred that the graphene blisters induced by anion intercalation in the first cycle enlarged during the de‐intercalation process and tended to destroy the lattice structure of the blister shell. However, the D peak weakened after the completion of the de‐intercalation process, likely indicating that the charge cut‐off voltage is not sufficiently high to completely break graphene blisters. In the second cycle, a typical phenomenon was observed that the blister was large at low voltage (larger D peak intensity) and small at high voltage (lower D peak intensity). Moreover, the activated graphene blisters possessed excellent elasticity, which was the basis for self‐repairing of “defects” after standing. Notably, the graphene blisters could be well recovered to the original state after the first discharge.

## 5 Prospective Method to Guide Graphite Cathode Design

The graphite cathodes in DIBs are inevitably associated with graphene blisters owing to the electrochemical oxidation process that graphite undergoes. Therefore, according to our analysis, a preparation method for graphene blisters with intact lattice and reversible deformation is a key factor for the structural design of graphite cathodes with large capacities. In addition to the electrochemical oxidation methods that have been widely reported in the literature, numerous other physical methods, such as electron irradiation, laser‐induced, and ultraviolet (UV) oxidation, that can be used to introduce the graphene blisters on graphite cathode are present.^[^ ^80^ ^]^

Johns et al. used in situ electron‐irradiation to treat nuclear graphite on a 200 kV transmission electron microscope with a dose rate and observed that the defect domains induced by electron‐irradiation underwent atomic rearrangement, which subsequently resulted in nanostructure formation in nuclear graphite.^[^ ^81^ ^]^ The hollow nanotube‐like defect could be observed in the graphite interlayer, and significant expansion in the *c* direction occurred between the basal planes. The authors referred to this lattice blister induced by high‐temperature electron‐irradiation as “localized swelling.” Additionally, the laser irradiation can be used to induce blistering. The interaction between the two neighboring graphene layers was governed by the balance between van der Waals forces and repulsive molecular orbital interactions. The laser pulse could supply the necessary energy required to overcome the interlayer cohesion without disrupting the in‐plane bonding.^[^ ^82^ ^]^ Therefore, the laser‐induced photoexfoliation technique was an effective method to form graphene blisters.^[^ ^83^ ^]^ Moreover, blister formation could be achieved via the combination of UV transparent anatase thin film and photocatalytic oxidation in the presence of water vapor.^[^ ^84^ ^]^ The gas‐assisted photo‐oxidation provided a novel photocatalytic method to uniformly oxidize the graphite surface. Oxidative gas species at reactive atomic carbon surface defects (i.e., GBs) caused graphite surface functionalization and the blistering of basal planes.^[^ ^85^ ^]^

**Figure 16:**
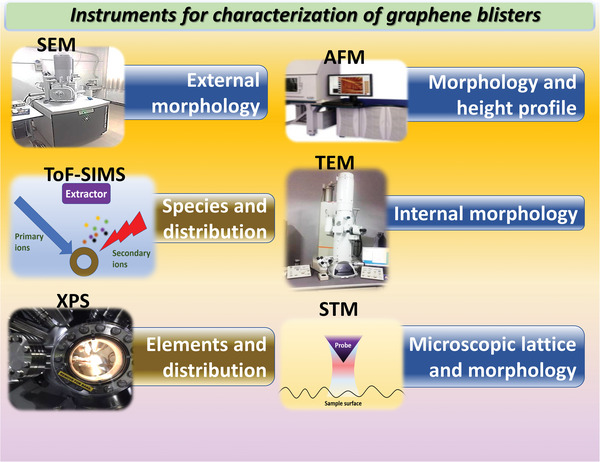
The list of instruments for characterization of graphene blisters.

It can be observed that graphite oxidation may induce blistering on the graphite surface, and in the meantime, the blister formation is often accompanied by intercalation of matters (ions or solvents, etc.) into graphite.^[^ ^86^ ^]^ Therefore, the anion intercalation rate can be well controlled in the electrochemical oxidation experiment, which is still the most effective method to induce blistering. The influences of the operating conditions, such as scan rate of CV, that control blister formation require further exploration.^[^ ^87^ ^]^ Moreover, how to characterize and detect graphene blisters is also important, which will affect our understanding of the properties of graphene blisters and how they can be used. Combined with the results of many references in this paper, we can know that several characterization methods play an important role in the study of graphene blisters, as listed in **Figure** **16**. These instruments will help us study the behavior of graphene blisters in DIBs. The morphology of the blister can be observed using SEM and AFM, while the latter can also be used to characterize the blister height. The distribution information of elements and species in the blister can be obtained through ToF‐SIMS and XPS technologies. Moreover, the internal morphology and the lattice parameter in the blister shell can be acquired by TEM and STM.

## 6 Conclusions and Outlook

DIBs use graphite, in which anions can intercalate, as the cathode material, and use the reversible intercalation/de‐intercalation process for secondary energy storage. During battery charging, the graphite cathode undergoes an electrochemical oxidation process, which can be viewed as a competition between the accommodation of anion inside graphite lattice and structural exfoliation of graphite. The researchers focused more attention on the composition of the electrolyte and the structural optimization of graphite cathodes in previous studies on DIBs. However, a special interfacial phenomenon has been neglected in battery research. Anion intercalation induces an intermediate state that is neither fully exfoliated nor initial surface—graphene blister—which was first discovered in the field of electrochemical oxidation of graphite. The intercalation of solvated anions enables 5–12 graphitic layers on the surface to separate from the bulk graphite and form a micro/nano‐sized semi‐circular protrusion, that is, graphene blister. Once the blister is formed, the blister with the shell containing several graphene layers exhibits unusual membrane‐like elastic behavior, which allows the graphene blister to remain its shell structure when it undergoes elastic deformation through reversible expansion and contraction. Although graphene blisters in DIBs have received little attention, the results of the Raman spectroscopy of graphene blisters in the field of electrochemical oxidation are almost the same those of in situ Raman spectrum changes during the cyclic charge/discharge process in DIBs. Therefore, it is known that graphene blisters can have favorable reversibility by regulating the cut‐off voltage, anionic components, and solvation. Moreover, we applied the “blister model” of graphene blisters to explain numerous anomalies in DIBs, and learn how the results from many different research fields correlate with each other, including the experimental or theoretical results from the electrochemical oxidation of graphite, electrowetting, DIBs, condensed matter physics, graphene bending nano‐mechanics, etc. Moreover, by reviewing the literatures we provide an expected physico‐electrochemical process: When the anions intercalate into graphite, the anions preferentially enter the lattice through the graphite GBs, producing graphene blisters with super elastic stiffness (5–12 graphitic layers in the shell); during anion de‐intercalation, the graphene blister enlarge owing to the absorption of the surrounding de‐intercalated solvated anions. The de‐intercalated anions will be attracted into the blister owing to the strong blister elasticity instead of returning to the bulk electrolyte immediately, which may be affected by the Marangoni effect.

In addition, numerous studies have shown that the formation of such graphene blisters can significantly increase the specific capacity of DIBs, but the formation and energy storage mechanism of graphene blisters in DIBs has rarely been researched. Therefore, we provide our expectations for graphene blisters: i) Highly reversible elastic deformation of graphene blisters for anion storage can be achieved by regulating the cut‐off voltage, anion, and solvent composition, or other physical oxidation methods; ii) Thin‐layer graphite with dense graphene blisters may significantly improve the specific capacity. However, several unresolved issues remain: i) After graphene blisters formation, the special physical and chemical properties of the blister shell, such as whether it has the properties of the selective permeation membrane, require to be further studied; ii) We have further predicted that a few solvated anions can be propelled via the Marangoni effect of graphene blisters, but this specific physical process requires to be characterized using advanced instruments; iii) The shape, pressure, cracking limit, and size of the graphene blisters are related to the electrolyte composition, so it is required to determine the appropriate solvent and the composition of the solvation shell of anion; iv) Self‐discharge rate of graphene blister should be studied in‐depth. Moreover, whether the inside of the graphene blister is equivalent to a minute electrolytic cell needs to be experimentally confirmed, which can hinder the outward diffusion of anions, if confirmed, significantly shortening the anion diffusion distance. We hope that graphene in graphite can bring fundamental changes and provide valuable insights into the research on cathode materials for DIBs in the future.

## Conflict of Interest

The authors declare no conflict of interest.

## References

[1] a) R. Hou , B. Liu , Y. Sun , L. Liu , J. Meng , M. D. Levi , H. Ji , X. Yan , Nano Energy 2020, 72, 104728;

[2] a) H. Wang , Y. Wang , Q. Wu , G. Zhu , Mater. Today 2022, 52, 269;

[3] a) J. Hao , X. Li , X. Song , Z. Guo , EnergyChem 2019, 1, 100004;

[4] W. Rüdorff , U. Hofmann , Z. Anorg. Allg. Chem. 1938, 238, 1.

[5] N. Bartlett , B. McQuillan , A. S. Robertson , Mater. Res. Bull. 1978, 13, 1259.

[6] a) D. Billaud , A. Pron , V. F. Lincoln , Synth. Met. 1980, 2, 177;

[7] a) Z. D. Wang , M. Inagaki , Synth. Met. 1988, 25, 181;

[8] K. S. Mohandas , N. Sanil , M. Noel , P. Rodriguez , Carbon 2003, 41, 927.

[9] C. Yang , J. Chen , X. Ji , T. P. Pollard , X. Lü , C. Sun , S. Hou , Q. Liu , C. Liu , T. Qing , Y. Wang , O. Borodin , Y. Ren , K. Xu , C. Wang , Nature 2019, 569, 245. 31068723 10.1038/s41586-019-1175-6

[10] R. T. Carlin , H. C. De Long , J. Fuller , P. C. Trulove , J. Electrochem. Soc. 1994, 141, L73.

[11] M. Yoshio , H. Nakamura , H. Wang , Electrochem. Solid‐State Lett. 2006, 9, A561.

[12] T. Placke , O. Fromm , S. F. Lux , P. Bieker , S. Rothermel , H. W. Meyer , S. Passerini , M. Winter , J. Electrochem. Soc. 2012, 159, A1755.

[13] H. Wang , M. Yoshio , Chem. Commun. 2010, 46, 1544. 10.1039/b914931g 20162176

[14] J. Gao , M. Yoshio , L. Qi , H. Wang , J. Power Sources 2015, 278, 452.

[15] a) T. Minato , T. Abe , Prog. Surf. Sci. 2017, 92, 240;

[16] a) W. H. Li , Y. M. Li , X. F. Liu , Z. Y. Gu , H. J. Liang , X. X. Zhao , J. Z. Guo , X. L. Wu , Adv. Funct. Mater. 2022, 32, 2201038;

[17] a) W. H. Li , H. J. Liang , X. K. Hou , Z. Y. Gu , X. X. Zhao , J. Z. Guo , X. Yang , X. L. Wu , J. Energy Chem. 2020, 50, 416;

[18] D. Yu , Q. Zhu , L. Cheng , S. Dong , X. Zhang , H. Wang , N. Yang , ACS Energy Lett. 2021, 6, 949.

[19] a) W. H. Li , X. L. Wu , Electrochem. Sci. Adv. 2021, 2, e2100127;

[20] a) Z. Siroma , K. Ishii , K. Yasuda , Y. Miyazaki , M. Inaba , A. Tasaka , Electrochem. Commun. 2005, 7, 1153;

[21] a) G. Bussetti , R. Yivlialin , D. Alliata , A. Li Bassi , C. Castiglioni , M. Tommasini , C. S. Casari , M. Passoni , P. Biagioni , F. Ciccacci , L. Duò , J. Phys. Chem. C 2016, 120, 6088;

[22] a) A. Ejigu , L. W. L. Fevre , K. Fujisawa , M. Terrones , A. J. Forsyth , R. A. W. Dryfe , ACS Appl. Mater. Interfaces 2019, 11, 23261; 31252480 10.1021/acsami.9b06528

[23] T. Ishihara , Y. Yokoyama , F. Kozono , H. Hayashi , J. Power Sources 2011, 196, 6956.

[24] M. S. Dresselhaus , R. Kalish , in Ion Implantation (Eds: M. S. Dresselhaus , R. Kalish ), Springer Berlin Heidelberg, Berlin, Heidelberg 1992.

[25] T. Ishihara , Y. Yokoyama , T. Shimosaka , F. Kozono , H. Hayashi , Nanosci. Nanotechnol. Lett. 2012, 4, 182.

[26] M. T. Lusk , L. D. Carr , Phys. Rev. Lett. 2008, 100, 175503. 18518307 10.1103/PhysRevLett.100.175503

[27] J. Lu , A. H. C. Neto , K. P. Loh , Nat. Commun. 2012, 3, 823. 22569367 10.1038/ncomms1818

[28] C. Herbig , E. H. Åhlgren , U. A. Schröder , A. J. Martínez‐Galera , M. A. Arman , J. Kotakoski , J. Knudsen , A. V. Krasheninnikov , T. Michely , Phys. Rev. B 2015, 92, 085429.

[29] W. Wang , X. Ma , Z. Dai , S. Zhang , Y. Hou , G. Wang , Q. Li , Z. Zhang , Y. Wei , L. Liu , Adv. Mater. Interfaces 2022, 9, 2101939.

[30] a) F. P. Campana , H. Buqa , P. Novák , R. Kötz , H. Siegenthaler , Electrochem. Commun. 2008, 10, 1590;

[31] a) K. Zhang , M. Arroyo , Extreme Mech. Lett. 2017, 14, 23;

[32] A. C. Chu , J. Y. Josefowicz , G. C. Farrington , J. Electrochem. Soc. 1997, 144, 4161.

[33] D. C. Alsmeyer , R. L. McCreery , Anal. Chem. 1992, 64, 1528.

[34] J. Zhang , E. k. Wang , J. Electroanal. Chem. 1995, 399, 83.

[35] C. A. Goss , J. C. Brumfield , E. A. Irene , R. W. Murray , Anal. Chem. 1993, 65, 1378.

[36] K. W. Hathcock , J. C. Brumfield , C. A. Goss , E. A. Irene , R. W. Murray , Anal. Chem. 1995, 67, 2201.

[37] D. Alliata , R. Kötz , O. Haas , H. Siegenthaler , Langmuir 1999, 15, 8483.

[38] D. Alliata , P. Häring , O. Haas , R. Kötz , H. Siegenthaler , Electrochem. Commun. 1999, 1, 5.

[39] R. Yivlialin , G. Bussetti , L. Brambilla , C. Castiglioni , M. Tommasini , L. Duò , M. Passoni , M. Ghidelli , C. S. Casari , A. Li Bassi , J. Phys. Chem. C 2017, 121, 14246.

[40] a) A. W. Robertson , K. He , A. I. Kirkland , J. H. Warner , Nano Lett. 2014, 14, 908; 24422539 10.1021/nl404266k

[41] G. Wang , Z. Dai , J. Xiao , S. Feng , C. Weng , L. Liu , Z. Xu , R. Huang , Z. Zhang , Phys. Rev. Lett. 2019, 123, 116101. 31573244 10.1103/PhysRevLett.123.116101

[42] E. Khestanova , F. Guinea , L. Fumagalli , A. K. Geim , I. V. Grigorieva , Nat. Commun. 2016, 7, 12587. 27557732 10.1038/ncomms12587 PMC5007416

[43] N. Levy , S. A. Burke , K. L. Meaker , M. Panlasigui , A. Zettl , F. Guinea , A. H. C. Neto , M. Crommie , Science 2010, 329, 544. 20671183 10.1126/science.1191700

[44] S. P. Koenig , N. G. Boddeti , M. L. Dunn , J. S. Bunch , Nat. Nanotechnol. 2011, 6, 543. 21841794 10.1038/nnano.2011.123

[45] J. S. Bunch , M. L. Dunn , Solid State Commun. 2012, 152, 1359.

[46] Z. Zong , C. L. Chen , M. R. Dokmeci , K. T. Wan , J. Appl. Phys. 2010, 107, 026104.

[47] Z. Xia , V. Bellani , J. Sun , V. Palermo , Faraday Discuss. 2021, 227, 291. 33346768 10.1039/c9fd00123a

[48] A. S. Daniel , Z. Dai , P. Wang , A. C. Chavez , J. B. Christopher , R. Huang , N. s. Lu , Proc. Natl. Acad. Sci. U. S. A. 2018, 115, 7884. 30006468 10.1073/pnas.1801551115 PMC6077740

[49] M. Tripathi , A. King , G. Fratta , M. Meloni , M. Large , J. P. Salvage , N. M. Pugno , A. B. Dalton , ACS Omega 2018, 3, 17000. 31458322 10.1021/acsomega.8b02815 PMC6644256

[50] M. Liao , Z. Liu , H. Xiao , W. Chen , X. Han , T. Zhao , Y. Tian , Laser Phys. 2020, 30, 065106.

[51] a) Z. Huang , Y. Hou , T. Wang , Y. Zhao , G. Liang , X. Li , Y. Guo , Q. Yang , Z. Chen , Q. Li , L. Ma , J. Fan , C. Zhi , Nat. Commun. 2021, 12, 3106; 34035250 10.1038/s41467-021-23369-5 PMC8149852

[52] L. N. Wu , S. Y. Shen , Y. H. Hong , C. H. Shen , F. M. Han , F. Fu , X. D. Zhou , L. Huang , J. T. Li , S. G. Sun , ACS Appl. Mater. Interfaces 2019, 11, 12570. 30855934 10.1021/acsami.9b01572

[53] O. V. Sinitsyna , G. B. Meshkov , A. V. Grigorieva , A. A. Antonov , I. G. Grigorieva , I. V. Yaminsky , Beilstein J. Nanotechnol. 2018, 9, 407. 29515954 10.3762/bjnano.9.40 PMC5815287

[54] a) M. Dienwiebel , G. S. Verhoeven , N. Pradeep , J. W. M. Frenken , J. A. Heimberg , H. W. Zandbergen , Phys. Rev. Lett. 2004, 92, 126101; 15089689 10.1103/PhysRevLett.92.126101

[55] S. D. Rosa , P. Branchini , R. Yivlialin , L. Duò , G. Bussetti , L. Tortora , ACS Appl. Nano Mater. 2020, 3, 691.

[56] a) J. Lu , J. Yang , J. Wang , A. Lim , S. Wang , K. Loh , ACS Nano 2009, 3, 2367; 19702326 10.1021/nn900546b

[57] A. L. Mayer , B. V. E. Jose , J. T. Falkenberg , G. Autès , A. W. Cummings , D. Soriano , G. Li , M. Brandbyge , O. V. Yazyev , S. Roche , E. Y. Andrei , 2D Mater. 2016, 3, 031005.

[58] P. Simonis , C. Goffaux , P. A. Thiry , L. P. Biro , P. Lambin , V. Meunier , Surf. Sci. 2002, 511, 319.

[59] J. Cervenka , C. F. J. Flipse , J. Phys.: Conf. Ser. 2007, 61, 190.

[60] J. Xhie , K. Sattler , M. Ge , N. Venkateswaran , Phys. Rev. B 1993, 47, 15835. 10.1103/physrevb.47.15835 10005981

[61] B. Schnyder , D. Alliata , R. Kötz , H. Siegenthaler , Appl. Surf. Sci. 2001, 173, 221.

[62] G. Park , N. Gunawardhana , H. Nakamura , Y. S. Lee , M. Yoshio , J. Power Sources 2012, 199, 293.

[63] S. J. Rodríguez , A. E. Candia , M. C. G. Passeggi , E. A. Albanesi , G. D. Ruano , Phys. Chem. Chem. Phys. 2021, 23, 19579. 34524287 10.1039/d1cp01855h

[64] T. He , C. Zhang , A. Du , Chem. Eng. Sci. 2019, 194, 58.

[65] A. Mukhopadhyay , Y. Yang , Y. Li , Y. Chen , H. Li , A. Natan , Y. Liu , D. Cao , H. Zhu , Adv. Funct. Mater. 2019, 29, 1903192.

[66] Q. Liu , Y. Wang , X. Yang , D. Zhou , X. Wang , P. Jaumaux , F. Kang , B. Li , X. Ji , G. Wang , Chem 2021, 7, 1993.

[67] M. Inaba , Z. Siroma , Y. Kawatate , A. Funabiki , Z. Ogumi , J. Power Sources 1997, 68, 221.

[68] M. Inaba , Y. Kawatate , A. Funabiki , S. K. Jeong , T. Abe , Z. Ogumi , Electrochim. Acta 1999, 45, 99.

[69] O. Matsuoka , A. Hiwara , T. Omi , M. Toriida , T. Hayashi , C. Tanaka , Y. Saito , T. Ishida , H. Tan , S. Ono , S. Yamamoto , J. Power Sources 2002, 108, 128.

[70] H. Youb , T. Fukutsuka , K. Miyazaki , T. Abe , J. Appl. Electrochem. 2016, 46, 1099.

[71] Y. Yang , D. Tang , C. Zhang , Y. Zhang , Q. Liang , S. Chen , Q. Weng , M. Zhou , Y. Xue , J. Liu , J. Wu , Q. Cui , C. Lian , G. Hou , F. Yuan , Y. Bando , D. Golberg , X. Wang , Environ. Sci. 2017, 10, 979.

[72] Y. Huang , J. Li , H. Wang , ACS Appl. Energy Mater. 2019, 2, 4544.

[73] Y. Huang , H. Wang , J. Electrochem. Soc. 2019, 166, A3838.

[74] R. Yazami , I. V. Goncharova , V. N. Plakhotnik , presented at New Carbon Based Materials for Electrochemical Energy Storage Systems: Batteries, Supercapacitors and Fuel Cells , Dordrecht 2006, 2006.

[75] S. Y. Luchkin , S. A. Lipovskikh , N. S. Katorova , A. A. Savina , A. M. Abakumov , K. J. Stevenson , Sci. Rep. 2020, 10, 8550. 32444787 10.1038/s41598-020-65552-6 PMC7244741

[76] G. Zhang , M. Walker , P. R. Unwin , Langmuir 2016, 32, 7476. 27406680 10.1021/acs.langmuir.6b01506

[77] R. Yivlialin , L. Brambilla , A. Accogli , E. Gibertini , M. Tommasini , C. Goletti , M. Leone , L. Duò , L. Magagnin , C. Castiglioni , G. Bussetti , Appl. Surf. Sci. 2020, 504, 144440.

[78] M. Balabajew , H. Reinhardt , N. Bock , M. Duchardt , S. Kachel , N. Hampp , B. Roling , Electrochim. Acta 2016, 211, 679.

[79] Y. Li , B. Wang , W. Li , K. Xu , ACS Nano 2022, 16, 6145. 35315643 10.1021/acsnano.1c11616

[80] a) E. Pavoni , R. Yivlialin , C. H. Joseph , G. Fabi , D. Mencarelli , L. Pierantoni , G. Bussetti , M. Farina , RSC Adv. 2019, 9, 23156; 35514520 10.1039/c9ra04667d PMC9067255

[81] S. Johns , T. Poulsen , J. J. Kane , W. E. Windes , R. Ubic , C. Karthik , Carbon 2019, 143, 908.

[82] I. M. Oraiqat , PhD thesis , University of Michigan 2016.

[83] a) Y. Miyamoto , H. Zhang , D. Tománek , Phys. Rev. Lett. 2010, 104, 208302; 20867073 10.1103/PhysRevLett.104.208302

[84] N. R. Ostyn , S. P. Sree , J. Li , J. Y. Feng , M. B. J. Roeffaers , S. De Feyter , J. Dendooven , C. Detavernier , J. A. Martens , Catal. Sci. Technol. 2021, 11, 6724.

[85] N. R. Ostyn , B. Thijs , J. A. Steele , S. P. Sree , W. Wangermez , J. Teyssandier , M. M. Minjauw , J. Li , C. Detavernier , M. B. J. Roeffaers , S. De Feyter , J. A. Martens , Carbon 2021, 172, 637.

[86] a) Z. Ogumi , M. Inaba , Bull. Chem. Soc. Jpn. 1998, 71, 521;

[87] M. S. Jagadeesh , A. Calloni , I. Denti , C. Goletti , F. Ciccacci , L. Duò , G. Bussetti , Surf. Sci. 2019, 681, 111.

